# Mesenchymal Stem Cells Prevent SLC39A14‐Dependent Hepatocyte Ferroptosis through Exosomal miR‐16‐5p in Liver Graft

**DOI:** 10.1002/advs.202411380

**Published:** 2024-12-16

**Authors:** Zhizhao Deng, Weiqi Zeng, Yingxin Gao, Zhenyu Yang, Xinling Luo, Xianlong Li, Guoliang Sun, Erfeng Xiong, Fei Huang, Gangjian Luo, Ziqing Hei, Dongdong Yuan

**Affiliations:** ^1^ Department of Anesthesiology The Third Affiliated Hospital of Sun Yat‐Sen University Guangzhou Guangdong 510630 China

**Keywords:** ferroptosis, hepatic ischemia‐reperfusion injury, mesenchymal stem cells, MiR‐16‐5p, SLC39A14

## Abstract

Ischemia‐reperfusion injury (IRI) is the leading cause of hepatic graft dysfunction, resulting from hepatocyte damage. Nevertheless, given the few specialized therapeutics available in hepatic IRI, additional mechanistic insights into hepatocyte damage are required. Here, the protein solute carrier family 39 member 14 (SLC39A14) is identified as a pro‐ferroptosis target in hepatocytes of human liver allografts through single‐cell RNA sequencing analysis. SLC39A14 knockdown significantly mitigated hepatic IRI by preventing hepatocyte ferroptosis in vivo and in vitro. Mechanistically, the inhibition of SLC39A14 suppressed non‐transferrin‐bound iron (NTBI) uptake by hepatocytes, thereby reducing iron overload and cell ferroptosis. Moreover, human bone marrow‐derived mesenchymal stem cells (hBMSCs) are found to exhibit a notable therapeutic effect on hepatic IRI by downregulating SLC39A14 expression. Exosomes derived from hBMSCs delivered abundant miR‐16‐5p into hepatocytes, which post‐transcriptionally suppressed the expression of SLC39A14 and reduced cell ferroptosis induced by hepatic IRI. In conclusion, SLC39A14 triggers hepatic IRI by mediating NTBI uptake into hepatocytes and inducing hepatocyte ferroptosis. Moreover, hBMSC‐based therapy is promising to reverse this progression of hepatic IRI.

## Graphical abstract

In hepatic IRI, SLC39A14 is identified as a driver of hepatocyte ferroptosis through facilitating extracellular NTBI transported into hepatocytes. Furthermore, hBMSCs has a significant therapeutic effect against hepatic IRI. hBMSCs delivered exosomal miR‐16‐5p, which can specifically target the 3'UTR region of SLC39A14 mRNA, thereby inhibiting SLC39A14‐mediated ferroptosis in liver graft.

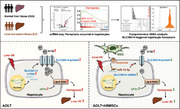

## 1 Introduction

Liver transplantation (LT) is susceptible to the risk of ischemia‐reperfusion injury (IRI). Severe IRI is known to be associated with early liver graft dysfunction and may require retransplantation.^[^ ^1^ ^]^ Although the clinical regimen for IRI in liver grafts has been improved, symptomatic treatment remains the main option^[^ ^2^ ^]^ and the progress of effective targeted therapies is constrained. Therefore, the precise molecular mechanisms underlying its pathogenesis still needs to be elucidated.

Hepatocellular injury plays a pivotal role in hepatic IRI, with hepatocytes, the predominant liver parenchymal cells, being highly vulnerable to both the cold/warm ischemia stage and the reperfusion stage. Multiple cell death pathways, such as ferroptosis, apoptosis, necrosis, and necroptosis, have been implicated in hepatocyte damage during hepatic IRI.^[^ ^3^ ^]^ However, in this study, ferroptosis was the most significant cell death pathway found in the hepatocytes of human liver grafts using single‐cell RNA sequencing, as opposed to apoptosis, controlled necrosis, pyroptosis, mitophagy, and autophagy. Several clinical and experimental investigations have shown the critical role ferroptosis plays in hepatic IRI.^[^ ^4^, ^5^, ^6^ ^]^ Different from other types of cell death, ferroptosis is characterized by the accumulation of iron overload and lipid peroxidation (LPO), accompanied by compromised antioxidant capacity. Of note, iron overload was reported to generate reactive oxygen species (ROS) through the Fenton reaction and enhance the activity of lipid synthesis‐related enzymes, which is the prerequisite of ferroptosis.^[^ ^7^ ^]^ Our previous study indicated that hepatic IRI appeared more severe in mice fed with high‐iron diet, whereas treatment with iron chelator deferoxamine could significantly alleviate hepatic IRI in mice fed with normal diet.^[^ ^8^ ^]^ A recent study also verified that iron overload could be positively correlated with the progression of hepatic IRI.^[^ ^9^ ^]^ These results indicate that iron overload plays a pivotal role in hepatic IRI. Furthermore, prior research has concentrated on the function of transferrin (TF)‐bound iron in hepatic IRI, which could be transported into hepatocytes by TF and subsequently induce iron overload.^[^ ^10^ ^]^ TF‐bound iron has long been recognized as a crucial element in sustaining cellular functionality. In contrast to TF‐bound iron, recent research suggested that non‐TF‐bound iron (NTBI), representing the residual iron not bound to TF, can exert detrimental effects on cellular function. For instance, NTBI‐induced iron overload has been reported to worsen vascular function and contribute to atherosclerosis.^[^ ^11^ ^]^ However, the exact molecular mechanisms through which iron overload occurs in hepatic IRI, particularly the involvement of NTBI, remain largely elusive.

Mesenchymal stem cells (MSCs)‐based therapy is currently regarded as one of the most promising strategies for protecting IRI in solid organ because of its high immunomodulation ability, tropism toward inflamed and injured tissues, and its widespread utilization.^[^ ^12^ ^]^ To date, over 1000 clinical trials involving MSCs have been registered in ClinicalTrials.gov, with up to 30 applied in LT. Experimental studies have demonstrated the remarkable therapeutic effect of MSCs on hepatic IRI and have preliminarily explored its functional mechanisms.^[^ ^13^, ^14^, ^15^ ^]^ However, the molecular mechanisms of MSCs in modulating the progression of IRI in liver grafts are complex and need to be fully elucidated. This represents a pivotal challenge that must be addressed to highlight the applicability and safety of MSC‐based therapy in hepatic IRI. The secretome from MSCs, particularly exosomes, which are extracellular vesicles enriched in mRNAs or microRNAs (miRNAs), is known to play a role in immunomodulation and inflammatory regulation.^[^ ^16^ ^]^ Exosomal miRNAs derived from MSCs interact with the 3′untranslated region of the target mRNA sequences, suppressing mRNA translation and mediating therapeutic effects on diseases. For instance, miR‐20a, miR‐1246, and miR‐124‐3p derived from MSCs have been reported to protect hepatocytes against hepatic IRI by modulating apoptosis, autophagy, and ferroptosis in hepatocytes.^[^ ^17^ ^]^ Given the diversity of exosomal miRNA derived from MSCs, further exploration into the functions of their different types in the context of IRI in liver grafts is needed.

The present study aimed to investigate the precise mechanism underlying iron overload and ferroptosis in hepatic IRI and to further explore the potential therapeutic impact of MSC‐based therapy on hepatic IRI.

## 2 Results

### 2.1 Silencing SLC39A14 Inhibits Hepatocyte Ferroptosis and Alleviates Hepatic Ischemia and Reperfusion Injury

**Figure 1:**
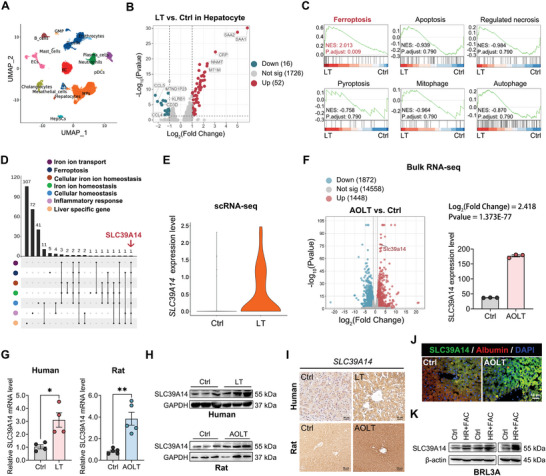
SLC39A14 is linked with hepatocyte ferroptosis and its expression is increased in liver graft. A) The uniform manifold approximation and projection (UMAP) plot of the overall identified clusters. B) Differentially expressed genes in hepatocyte cluster by scRNA‐seq analysis. C) Gene set enrichment analysis (GSEA) revealing the enrichment of terms in curated gene sets (C2) in hepatocyte cluster by scRNA‐seq analysis. D) Upset plot showing gene overlap analysis of the specific GO terms in hepatocyte cluster by scRNA‐seq analysis. E) SLC39A14 expression level in hepatocyte cluster by scRNA‐seq analysis. F) Differentially expressed genes and SLC39A14 mRNA level in liver tissue by bulk RNA‐seq. Data are shown as mean ± SD, *n* = 3. G) RT‐qPCR was used to detect SLC39A14 mRNA level in liver tissue from human (*n* = 4) and rats (*n* = 5). Data are shown as mean ± SEM. H‐K) Western blotting, immunohistochemistry, and immunofluorescence assay were used to detect SLC39A14 protein level in liver tissue from humans and rats as well as in BRL3A cells. Data are shown as mean ± SEM, *n* = 5. *p < 0.05, **p < 0.01.

To identify the features of hepatocyte injury in hepatic IRI, scRNA‐seq analysis on human normal liver specimens and liver grafts was performed (Figure S1A, Supporting Information). The quality control of gene expression in each cell is shown in Figure S1B (Supporting Information). The gene expression profiles of liver cells from both normal liver specimens and liver grafts were categorized into 16 clusters, among which hepatocyte cluster was characterized by *ALB*+, *APOB*+, *APOC1*+, and *HP*+, while being negative for *EPCAM*‐ and *KRT19*‐ (**Figure** **1**A; Figure S1C, Supporting Information). In hepatocyte cluster, 1794 DEGs were identified, of which 52 were significantly upregulated (Log_2_FC > 1 and *p* < 0.05) and 16 were significantly downregulated (Log_2_FC < −1 and *p* < 0.05) (Figure 1B). Analysis on the DEGs by GSEA revealed that ferroptosis was the prominent cell death form significantly enriched in hepatocytes rather than others, such as apoptosis, regulated necrosis, pyroptosis, mitophagy, and autophagy (Figure 1C). GO terms related to oxidative stress, lipid synthesis, lipid transport, lipid oxidation, and iron ion homeostasis as well as iron transport were all highly enriched in hepatocytes from LT group (Figure S1D–F, Supporting Information). The results above indicate that cell ferroptosis may play an important role in hepatic IRI and was selected for further studies. First, we found that 4‐HNE and GPX4, two classic markers of cell ferroptosis, were significantly upregulated and downregulated in human liver grafts and in rats with AOLT, respectively (Figure S2A,B, Supporting Information). TEM results showed that mitochondrial crista was severely destructed or disappeared, and the mitochondrial membrane density was increased in human liver grafts and in rats with AOLT (Figure S2A,B, Supporting Information). Additionally, the levels of MDA and Fe^2+^ were much higher in rats with AOLT (Figure S2C, Supporting Information). Then, we found that the canonical ferroptosis inhibitor, Fer‐1, significantly decreased the levels of 4‐HNE, MDA, and Fe^2+^, and meanwhile up‐regulated GPX4 protein expression, thereby rescued hepatocellular damage in rats with AOLT (Figure S2B,E, Supporting Information). The anti‐ferroptosis effect of Fer‐1 was also observed in BRL3A cells treated with HR+FAC, and Fer‐1 effectively reduced cell damage induced by HR+FAC (Figure S2F–H, Supporting Information). These results confirm that ferroptosis occurs in hepatocytes and may play a crucial role in worsening liver graft injury.

Regarding the tight relationship between iron overload and cell ferroptosis,^[^ ^9^ ^]^ it was attempted to explore which gene mainly regulates iron overload in hepatocytes in hepatic IRI. Specifically, concentration was placed on the GO terms associated with cellular iron ion homeostasis, inflammatory response, and liver specific gene from GSEA analysis. Intriguingly, the divalent metal transporter, SLC39A14, was the only overlapped gene among these biological processes and was dramatically up‐regulated in LT group (Figure 1D,E), suggesting that SLC39A14 may relate to the iron overload in hepatocyte ferroptosis. Consistently, bulk RNA‐seq and further validation revealed that the RNA and protein level were significantly elevated in liver grafts from human and rats and in HR‐FAC‐treated BRL3A cells (Figure 1F–K). Notably, these changes were accompanied by poor hepatocellular injury (Figures S3 and S4, Supporting Information).

**Figure 2:**
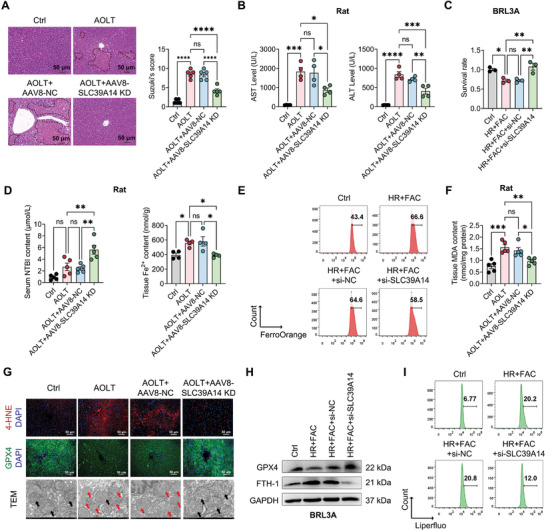
Knockdown of SLC39A14 reduces hepatocyte ferroptosis and alleviates hepatic ischemia and reperfusion injury. A, B) Evaluation of liver graft injury. A) Pathological analysis by H&E staining and Suzuki's score. Data are shown as mean ± SD, *n* = 5. B) Serum AST and ALT concentration. Data are shown as mean ± SD, *n* = 4. C) CCK8 assessed cell damage severity in BRL3A cells. Data are shown as mean ± SEM, *n* = 3. D) Levels of non‐transferrin‐bound iron (NTBI, left, *n* = 5) in serum and ferrous iron (Fe^2+^, right, *n* = 4) in liver tissues from rats. Data are shown as mean ± SD. E) Flow cytometry analysis of ferrous iron (Fe^2+^) level in BRL3A cells. F) Level of MDA in liver tissue from rats. Data are shown as mean ± SD, *n* = 5. G) Immunofluorescence staining showing the expression levels of 4‐HNE and GPX4 protein and transmission electron microscopy (TEM) showing the mitochondrial morphology of hepatocytes in liver tissue from rats (black arrow: normal mitochondrion; red arrow: the damaged mitochondrion). H) Western blotting showing levels of GPX4 and FTH‐1 protein in BRL3A cells. I) Flow cytometry analysis of lipid peroxidation level in BRL3A cells. *p < 0.05, **p < 0.01, ***p < 0.001, ****p < 0.0001. HR+FAC, hypoxia and reperfusion with ferric ammonium citrate.

We next evaluated the function of SLC39A14 in hepatocyte ferroptosis by knocking down its expression in vivo and in vitro. As shown, AAV8 vectors carrying with a liver‐specific promoter and *SLC39A14* shRNA were primarily transduced into the liver tissue rather than the kidney, lung, or spleen in rats (Figure S5A,B, Supporting Information). When SLC39A14 expression was successfully silenced, hepatocellular injury was significantly rescued in AOLT rats and BRL3A cells treated with HR+FAC (**Figure** **2**A–C; Figure S5C,D, Supporting Information). SLC39A14 has been reported to mediate the uptake of NTBI in a way of Fe^2+^, resulting in intracellular iron overload.^[^ ^18^ ^]^ In this study, increased Fe^2+^ content in liver tissues was found in AOLT rats, however, knockdown of SLC39A14 significantly reversed this change accompanied with a prominent elevation in serum NTBI level (Figure 2D). Consistently, silencing of SLC39A14 also dramatically suppressed the increase of intracellular Fe^2+^ level in BRL3A cells caused by HR+FAC (Figure 2E). These results suggested that SLC39A14 resulted in iron overload probably through mediating NTBI into hepatocytes. Importantly, knockdown of SLC39A14 could effectively alleviate hepatocyte ferroptosis in AOLT rats and HR+FAC‐treated BRL3A cells, manifested by the decrease of MDA, 4‐HNE, LPO, and FTH‐1 protein level, and the increase of GPX4 protein level as well as milder mitochondrion damage (Figure 2F–I). Collectively, the abovementioned findings indicate that knockdown SLC39A14 inhibits iron overload in hepatocytes caused by NTBI uptake, thereby alleviating hepatocyte ferroptosis and liver graft injury.

### 2.2 hBMSCs Inhibit SLC39A14 Expression through Releasing Exosomes to Reduce Hepatocyte Ferroptosis and Alleviate Hepatic Ischemia and Reperfusion Injury

**Figure 3:**
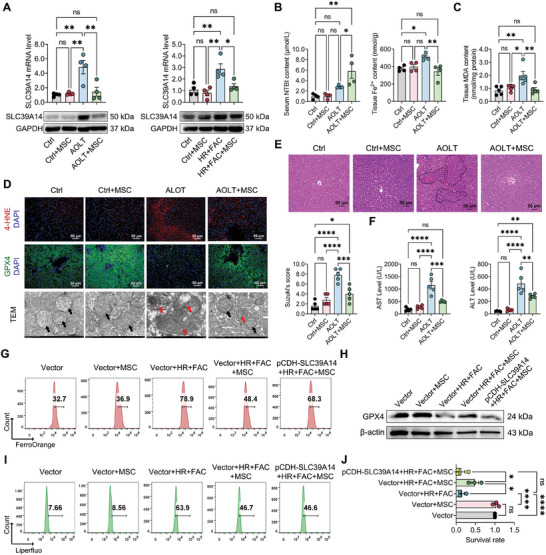
hBMSCs inhibit SLC39A14 expression to reduce hepatocyte ferroptosis and alleviate hepatic ischemia and reperfusion injury. A) RT‐qPCR and western blotting detecting mRNA and protein levels of SLC39A14 in liver tissue from rats and BRL3A cells. Data are shown as mean ± SEM, *n* = 4. B) Levels of non‐transferrin‐bound iron (NTBI, left) in serum and ferrous iron (Fe^2+^, right) in liver tissues from rats. Data are shown as mean ± SD, *n* = 4. C) Level of MDA in liver tissue from rats. Data are shown as mean ± SD, *n* = 5. D) Immunofluorescence staining showing the expression levels of 4‐HNE and GPX4 protein and transmission electron microscopy (TEM) showing the mitochondrial morphology of hepatocytes in liver tissue from rats (black arrow: normal mitochondrion; red arrow: the damaged mitochondrion). (E, F) Evaluation of liver graft injury. E) Pathological analysis by H&E staining and Suzuki's score. Data are shown as mean ± SD, *n* = 5. F) Serum AST and ALT concentration. Data are shown as mean ± SD, *n* = 5. G‐J) pCDH‐3xflag‐NC or SLC39A14‐overexpressed BRL3A cells were co‐cultured with or without MSCs and treated with or without HR+FAC. G) Flow cytometry analysis of ferrous iron (Fe^2+^) level in BRL3A cells. (H) Western blotting showing levels of GPX4 protein in BRL3A cells. (I) Flow cytometry analysis of lipid peroxidation level in BRL3A cells. (J) CCK8 assessed cell damage severity in BRL3A cells. Data are shown as mean ± SEM, *n* = 3. *p < 0.05, **p < 0.01, ***p < 0.001, ****p < 0.0001. HR+FAC, hypoxia, and reperfusion with ferric ammonium citrate. MSC, mesenchymal stem cell.

hBMSCs have been found exhibiting beneficial properties in IRI following solid organ transplantation.^[^ ^19^ ^]^ However, the mechanism behind these effects remains largely unknown, prompting exploration into whether hBMSCs could influence SLC39A14 expression level to improve liver graft injury. First, we observed that numerous hBMSCs were prone to stay in liver tissue 24 h following injection via the portal vein (Figure S6, Supporting Information). Intriguingly, pretreating with hBMSCs significantly lowered SLC39A14 RNA and protein levels in AOLT rats and HR+FAC‐treated BRL3A cells (**Figure** **3**A; Figure S7A, Supporting Information), suggesting that hBMSCs potentially regulated SLC39A14 expression in liver grafts. In AOLT rats, as SLC39A14 level was reduced by hBMSCs, Fe^2+^ overload in liver was significantly eliminated and serum NTBI level was followingly elevated (Figure 3B). Meanwhile, the ferroptosis manifestations, indicated by 4‐HNE and GPX4 level as well as mitochondria injury, were apparently rescued in AOLT rats treated with hBMSCs (Figure 3D). Consistently, hBMSCs effectively reduced hepatocyte damage in AOLT rats and HR+FAC‐treated BRL3A cells (Figure 3E,F). These results suggest that hBMSCs may exert an anti‐ferroptosis effect on liver graft through inhibiting SLC39A14‐mediated NTBI transport.

To further examine the protective effect of hBMSCs was related to its inhibition on SLC39A14 function, we generated a BRL3A cell line stably expressing SLC39A14 (Figure S7B, Supporting Information). The results showed that hBMSCs did not decrease the intracellular Fe^2+^ level in SLC39A14‐overexpressing BRL3A cells upon HR+FAC (Figure 3G). In addition, the anti‐ferroptosis effect brought by hBMSCs was dramatically offset in SLC39A14‐overexpressing BRL3A cells treated with HR+FAC, manifested by low level of GPX4 protein but high level of LPO (Figure 3H,I; Figure S7C, Supporting Information). Finally, even treating with hBMSCs, SLC39A14‐overexpressing BRL3A cells still exhibited a poor survival rate upon HR+FAC (Figure 3J). These findings add more evidence that hBMSCs inhibit SLC39A14 expression to rescue liver graft injury.

**Figure 4:**
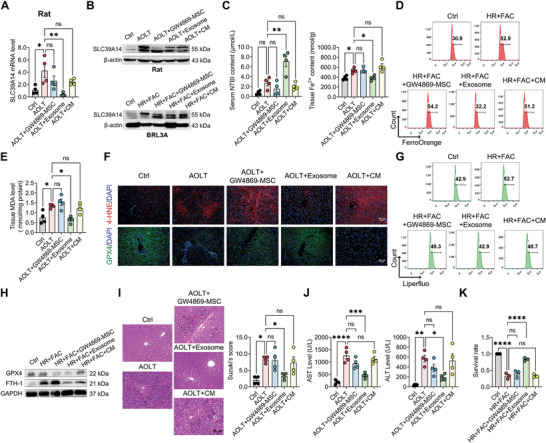
hBMSCs exserts therapeutic effects on hepatic ischemia and reperfusion injury through exosomes. A) Level of SLC39A14 mRNA in liver tissues from rat. Data are shown as mean ± SEM, *n* = 4. B) Westen blotting detecting SLC39A14 protein level in liver tissue from rats and in BRL3A cells. C) Levels of non‐transferrin‐bound iron (NTBI, left) in serum and ferrous iron (Fe^2+^, right) in liver tissues from rats. Data are shown as mean ± SD, *n* = 4. D) Flow cytometry analysis of ferrous iron (Fe^2+^) level in BRL3A cells. E) Level of MDA in liver tissue from rats. Data are shown as mean ± SD, *n* = 4. F) Immunofluorescence staining showing the expression levels of 4‐HNE and GPX4 protein in liver tissue from rats. G) Flow cytometry analysis of lipid peroxidation level in BRL3A cells. H) Western blotting showing levels of GPX4 and FTH‐1 protein in BRL3A cells. I, J) Evaluation of liver graft injury. (I) Pathological analysis by H&E staining and Suzuki's score. Data are shown as mean ± SD, *n* = 5. (J) Serum AST and ALT concentration. Data are shown as mean ± SD, *n* = 4. K) CCK8 assessed cell damage severity in BRL3A cells. Data are shown as mean ± SEM, *n* = 3. *p < 0.05, **p < 0.01, ***p < 0.001, ****p < 0.0001. HR+FAC, hypoxia and reperfusion with ferric ammonium citrate.

Exosomes have been recognized as the important intercellular communication mode of MSCs,^[^ ^20^ ^]^ and therefore, it was attempted to investigate whether exosomes were the functional substances in hBMSCs on liver graft injury. The characteristics of exosomes derived from hBMSCs are presented in Figure S8A–D (Supporting Information). We first observed that GW4869, a known inhibitor of exosome synthesis and release,^[^ ^21^ ^]^ significantly abated the inhibitory effect of hBMSCs on SLC39A14 expression in AOLT rats and HR+FAC‐treated BRL3A cells (**Figure** **4**A,B; Figure S9A,B, Supporting Information). MSC pretreated with GW4869 did not decrease the Fe^2+^ content (Figure 4C,D), inhibit the production of lipid peroxidation (Figure 4E–G) and rescue the expression of anti‐oxidative enzyme GPX4 (Figure 4F,H; Figure S9C, Supporting Information) in AOLT rats and HR+FAC‐treated BRL3A cells. Together, MSC pretreated with GW4869 failed improving the hepatocyte damage in AOLT rats and HR+FAC‐treated BRL3A cells (Figure 4I–K), suggesting that hBMSCs may benefit liver graft injury through exosomes. Next, we isolated exosomes from hBMSCs and found that exosomes also significantly reduced SLC39A14 expression as same as hBMSCs rather than conditioned medium with exosomes removed (CM) in AOLT rats and HR+FAC‐treated BRL3A cells (Figure 4A,B; Figure S9A,B, Supporting Information). Consistently, exosomes not only lowered intracellular Fe^2+^ level (Figure 4C,D), but also rescued hepatocyte ferroptosis (Figure 4E–H; Figure S9C, Supporting Information) and cell damage (Figure 4I–K) in AOLT rats and HR+FAC‐treated BRL3A cells, while CM showing no therapeutic effects as exosomes (Figure 4C–K). Overall, the results indicated that the protective effect of hBMSCs on liver graft injury may be dependent on exosomes‐based intercellular communication.

### 2.3 miR‐16‐5p Emerges as the Functional Molecule in hBMSCs‐Derived Exosomes in Reducing Hepatic Ischemia and Reperfusion Injury

**Figure 5:**
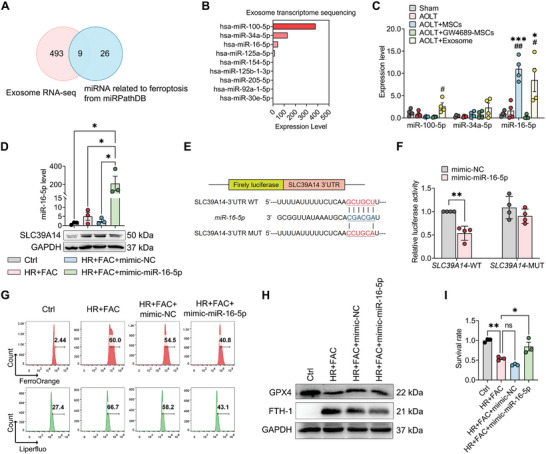
miR‐16‐5p emerges as the functional molecule in hBMSCs‐derived exosomes in reducing hepatic ischemia and reperfusion injury. A) Venn plot showing the number of overlapping genes between RNA‐seq analysis and ferroptosis‐related miRNAs in miRPathDB database. B) Bar plot showing the expression levels of overlapping genes by RNA‐seq. C) RT‐qPCR detecting the expression levels of miR‐16‐5P, miR‐100‐5p, and miR‐34a‐5p in liver tissue from rats. Data are shown as mean ± SEM, *n* = 4. D) RT‐qPCR detecting miR‐16‐5p level in BRL3A cells. Data are shown as mean ± SEM, *n* = 3. E) Sequence alignments of miR‐16‐5p and its candidate target sites in the 3′UTR of SLC39A14. F) Luciferase reporter assay of miR‐16‐5p mimic‐treated HEK293T cells, which overexpressed either SLC39A14‐wildtype 3′UTR (WT) or SLC39A14‐mutant 3′UTR (MT). G) Flow cytometry analysis of ferrous iron (Fe^2+^) level and lipid peroxidation level in BRL3A cells. H) Western blotting detecting the expression levels of GPX4 and FTH‐1 protein in BRL3A cells. I) CCK8 assessed cell damage severity in BRL3A cells. Data are shown as mean ± SEM, *n* = 3. *p < 0.05, **p < 0.01, ***p < 0.001, ****p < 0.0001.

Recent evidence supported the therapeutic potential of exosomes, containing specific microRNAs (miRNAs), in the treatment of IRI.^[^ ^22^, ^23^ ^]^ To further study the mechanism underlying the protective effect of exosomes derived from hBMSCs, high‐throughput transcriptome sequencing was employed to profile miRNAs in hBMSC‐derived exosomes (hBMSC‐exosomes). The analysis revealed enrichment of 493 annotated miRNAs in hBMSC‐exosomes, of which nine belonged to the ferroptosis‐related miRNA set from miRPathDB (**Figure** **5**A). has‐miR‐100‐5p, has‐miR‐34a‐5p, and has‐miR‐16‐5p emerged as the top three miRNAs (Figure 5B). RT‐qPCR results showed that miR‐16‐5p was the most highly enriched miRNA in hBMSCs‐ or exosomes‐treated groups (Figure 5C). When BRL3A cells were transfected by miR‐16‐5p mimic, HR+FAC‐enhanced SLC39A14 protein expression was significantly reversed, suggesting that SLC39A14 was possibly one of the target genes of miR‐16‐5p (Figure 5D; Figure S11A, Supporting Information). This relationship was also predicted by starBase (ver. 2.0),^[^ ^24^ ^]^ including one predicted target site for miR‐16‐5p in the 3′ untranslated region (UTR) of the transcript of *SLC39A14* gene (Figure 5E; Figure S10, Supporting Information). To verify the direct binding of miR‐16‐5p to the 3′UTR region of SLC39A14 gene, it was attempted to clone the wild‐type (WT) and mutant (MUT) 3‐UTR of SLC39A14 downstream of a firefly luciferase cassette in a luciferase receptor vector. The results showed that co‐transfection of miR‐16‐5p mimic with the WT receptor plasmids in HEK‐293T cells could significantly reduce the luciferase activity, which was significantly reversed by co‐transfection with the MUT receptor plasmids (Figure 5F). These findings indicated that miR‐16‐5p from hBMSC‐exosomes can potentially bind the 3′UTR region of SLC39A14 mRNA, thereby inhibiting SLC39A14 expression through translational suppression.

Moreover, the miR‐16‐5p mimic significantly decreased hepatocellular Fe^2+^ overload, remarkedly reducing levels of LPO and FTH‐1 and restoring GPX4 expression in HR+FAC‐ treated BRL3A cells (Figure 5G,H; Figure S11B, Supporting Information). Higher level of miR‐16‐5p significantly improved hepatocyte cell death caused by HR+FAC (Figure 5I). These results suggest that miR‐16‐5p binds to *SLC39A14* gene and potentially inhibit its expression, alleviating hepatocyte ferroptosis and damage caused by HR.

**Figure 6:**
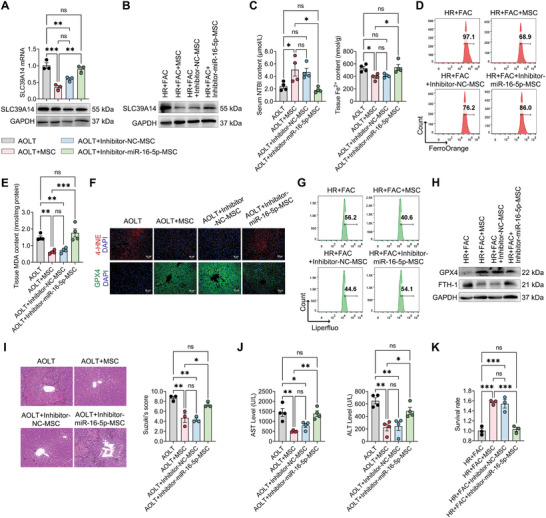
miR‐16‐5p targets SLC39A14 expression to inhibit hepatocyte ferroptosis and reduce hepatic ischemia and reperfusion injury. A) RT‐qPCR and western blotting detecting SLC39A14 mRNA and protein expression in liver tissue from rats. Data are shown as mean ± SEM, *n* = 3. B) Western blotting detecting SLC39A14 protein level in BRL3A cells. C) Levels of non‐transferrin‐bound iron (NTBI, left) in serum and ferrous iron (Fe^2+^, right) in liver tissues from rats. Data are shown as mean ± SD, *n* = 4. D) Flow cytometry analysis of ferrous iron (Fe^2+^) level in BRL3A cells. E) Level of MDA in liver tissue from rats. Data are shown as mean ± SD, *n* = 4. F) Immunofluorescence staining showing the expression levels of 4‐HNE and GPX4 protein in liver tissue from rats. G) Flow cytometry analysis of lipid peroxidation level in BRL3A cells. H) Western blotting showing levels of GPX4 and FTH‐1 protein in BRL3A cells. (I, J) Evaluation of liver graft injury. I) Pathological analysis by H&E staining and Suzuki's score. Data are shown as mean ± SD, *n* = 3. J) Serum AST and ALT concentration. Data are shown as mean ± SD, *n* = 4. (K) CCK8 assessed cell damage severity in BRL3A cells. Data are shown as mean ± SEM, *n* = 3. *p < 0.05, **p < 0.01, ***p < 0.001, ****p < 0.0001. HR+FAC, hypoxia, and reperfusion with ferric ammonium citrate.

To determine the function of miR‐16‐5p from hBMSCs on liver graft injury in AOLT rats or HR+FAC‐treated BRL3A cells, miR‐16‐5p inhibitor was transfected into hBMSCs. We observed that hBMSCs transfected with miR‐16‐5p inhibitor failed to downregulate the mRNA and protein expression of SLC39A14 in liver tissue from AOLT rats and in BRL3A cells (**Figure** **6**A,B; Figure S12A,B, Supporting Information). Additionally, as miR‐16‐5p exhibited ineffectiveness in hBMSCs, the capacity of hBMSCs to reduce tissue Fe^2+^ content in AOLT liver was significantly reversed accompanied by a notable increase in serum NTBI level (Figure 6C). Similarly, hBMSCs transfected with inhibitor‐miR‐16‐5p were unable to decrease intracellular Fe^2+^ level in HR+FAC‐treated BRL3A cells (Figure 6D). These results indicated that miR‐16‐5p plays an important role in regulating SLC39A14‐mediated NTBI uptake into hepatocytes in hBMSCs. Furthermore, inhibiting the function of miR‐16‐5p reduced the anti‐ferroptosis capability of hBMSCs in HIRI. This reduction was evident by increased lipid peroxidation products including 4‐HNE, MDA, and LPO and elevated FTH‐1 expression as well as impaired GPX4 expression (Figure 6E,H; Figure S12C,D, Supporting Information). Ultimately, owing to the weakened protective effect of hBMSCs upon miR‐16‐5p inhibitor, hepatocyte damage was still severe in AOLT rats and HR+FAC‐treated BRL3A cells (Figure 6I–K). Taken together, these findings provided primary evidence regarding the functional role of miR‐16‐5p in hBMSCs in inhibiting SLC39A14 expression, thereby protecting against ferroptosis‐induced HIRI.

## 3 Discussion

**Figure 7:**
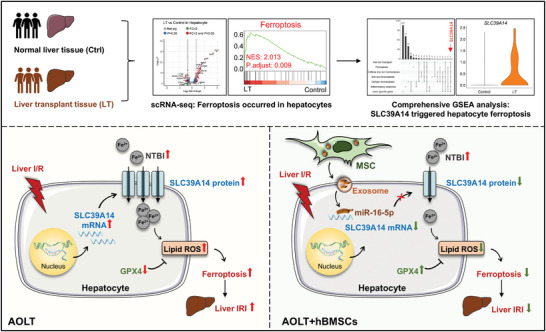
Graphical scheme of SLC39A14‐induced hepatocyte ferroptosis in hepatic ischemia and reperfusion injury and protective effect from MSCs‐derived exosomal miR‐16‐5p. scRNA‐seq, single cell RNA sequencing; IRI, ischemia and reperfusion injury; MSC, mesenchymal stem cells; SLC39A14, solute carrier family 39 member 14; GPX4, glutathione peroxidase 4.

Hepatic IRI is a long‐lasting clinical challenge that crucially impacts the prognosis of patients undergoing LT, and additional studies are therefore required to investigate the precise molecular mechanism by which hepatocyte damage occurs due to hepatic IRI and to identify the potential targeted therapies. In the present study, scRNA‐seq analysis revealed that hepatocyte ferroptosis played a critical role in hepatic IRI, with SLC39A14 being newly identified as a potential driver. It mediated the transport of extracellular NTBI into hepatocytes, thereby activating ferroptosis. Additionally, hBMSCs exhibited a significant therapeutic effect on hepatic IRI. Mechanistically, hBMSCs delivered exosomal miR‐16‐5p, which could specifically target the 3′UTR region of SLC39A14 mRNA, thereby suppressing SLC39A14‐mediated ferroptosis in liver graft (**Figure** **7**). This study not only provides a new mechanistic insight into iron overload in hepatocyte ferroptosis in liver graft, but also offers additional mechanistic evidence supporting the protective effect of hBMSCs on hepatic IRI.

In hepatic IRI, the hepatic cell death programs often exhibit an overlapping forms of regulated cell death. Various types of hepatocyte death, including apoptosis, necroptosis, pyroptosis and ferroptosis, have been found to participate in hepatic IRI.^[^ ^9^, ^25^, ^26^, ^27^ ^]^ Ferroptosis is a new form of cell death characterized by iron‐dependent peroxidation of unique lipid like PUFA‐PL‐OOH, as well as by the exhaustion of anti‐oxidant system.^[^ ^7^ ^]^ Although iron accumulation is unequal to the occurrence of ferroptosis, it is a prerequisite for ferroptosis. Extensive research has demonstrated that the removal of iron ions using iron chelators effectively impedes cell ferroptosis in hepatic, renal, cerebral, and cardiac IRI,^[^ ^28^, ^29^, ^30^ ^]^ indicating that targeting on iron metabolism is a promising approach to control ferroptosis. Consistent with the previous studies, the current study demonstrated that iron overload mainly occurs in the liver subjected to IRI, leading to detrimental effect in terms of substantial production of radials via the Fenton reaction. However, previous studies rarely assessed the precise mechanism by which iron could be accumulated in the liver subjected to IRI. In the present study, SLC39A14 emerged as a key gene in mediating iron overload and hepatocyte ferroptosis in liver graft based on scRNA‐seq analysis. SLC39A14 has been reported as a mental ion transporter for zinc, iron, and manganese, participating in the pathogenesis of different diseases. In a murine model of hereditary hemochromatosis, SLC39A14 was found essential in promoting hepatocellular iron overload.^[^ ^31^ ^]^ In addition, SLC39A14 could mediate NTBI uptake and cause intestinal injury and intestinal flora dysbiosis.^[^ ^32^ ^]^ Of note, in the present study, knockdown of SLC39A14 in AOLT rats significantly reversed the increased iron content in liver, while the serum iron level was notably higher compared to SLC39A14‐NC AOLT rats. Additionally, hepatocyte ferroptosis was markedly reduced, and SLC39A14‐KD rats exhibited milder liver injury after AOLT. These findings revealed that SLC39A14, at least in part, is responsible for iron overload in liver graft and contributes to the promotion of hepatocyte ferroptosis in hepatic IRI.

Iron is known for its crucial role in biological processes, necessitating the identification of the specific iron component closely associated with hepatocyte ferroptosis in liver graft injury. Typically, serum iron forms a complex with TF, and this complex is internalized into the cytoplasm through transferrin receptor (TFRC)‐mediated endocytosis. Minghui Gao and et al demonstrated that inhibiting TFRC expression level significantly suppressed cell ferroptosis, even in the presence of exogenously added APO‐TF under TFRC depletion conditions.^[^ ^33^ ^]^ Therefore, the uptake of TF‐bound iron was considered as the primary mechanism facilitating iron overload, ultimately triggering cell ferroptosis. However, under pathological circumstances where the serum TF level reaches saturation or is low, the serum NTBI level may significantly rise. It is widely accepted that serum NTBI level is rapidly and effectively absorbed by the liver.^[^ ^34^ ^]^ NTBI is recognized for catalyzing toxic reactions and inducing lipid peroxidation damage in cells,^[^ ^35^ ^]^ which aligns with the observed hepatocytes damage in both the human LT group and the AOLT rat group. As SLC39A14 has been demonstrated to mediate NTBI uptake into cells,^[^ ^18^ ^]^ the changes in serum NTBI and tissue Fe^2+^ contents in SLC39A14‐KD rats with AOLT were measured. In the present study, when SLC39A14 expression was significantly inhibited, the Fe^2+^ content in hepatocytes was dramatically decreased in AOLT rats. Notably, this change was accompanied by the increase of serum NTBI, suggesting that the elevated Fe^2+^ level in liver was probably facilitated by NTBI uptake. Our findings revealed that SLC39A14‐mediated NTBI transport could be the main cause of iron overload and hepatocyte ferroptosis in liver graft, providing a new mechanistic insight into iron overload in liver graft.

MSCs have exhibited a promising therapeutic effect on hepatic IRI, which has also been evidenced in both AOLT rat model and HR+FAC BRL3A cell model in this study. Numerous studies showed that MSCs have great potential to modulate inflammatory response and immune system response by producing extracellular vesicles (EVs), cytokines, and growth factors that can mediate cell‐to‐cell communications. Exosomes, a class of EVs ranging in size from 30 to 150 nm, have been recognized as one of the most important functional components in MSCs. They function as a bridge, facilitating the crucial transfer of non‐coding RNAs (ncRNAs), lipids, and membrane‐bound proteins from donor to recipient cells. The outcomes of GW4689 application (an inhibitor of exosome synthesis and release) application on hBMSCs in this study substantiated the finding that the therapeutic efficacy of hBMSCs is largely due to their secretion of paracrine factors in the form of exosomes. While we directly observed the uptake of Dil‐labeled exosomes by BRL3A cells in vitro and the accumulation of PKH26‐labeled exosomes in liver tissue in vivo, the identification of recipient cell types in rats requires further exploration. Furthermore, Xin et al. have emphasized the functional role of exosomal miRNA in the initiation and progression of solid organ IRI. Notably, miRNAs are small endogenous non‐coding RNAs, ≈20–24 nucleotides in length, known to regulate posttranscriptional gene expression by acting as negative gene regulators. Considering the diverse arrays of miRNAs, the regulatory miRNAs derived from MSC‐exosomes in hepatic IRI are still largely unknown. Through transcriptomic sequencing analysis and RT‐qPCR verification, a novel regulatory RNA, miR‐16‐5p, was identified as one of the functional miRNAs in hBMSCs. Specifically, treatment with a miR‐16‐5p inhibitor significantly reversed the therapeutic effects of hBMSCs. Numerous studies have explored the beneficial effects of miR‐16‐5p on cancer therapy, burn wound healing, and the inhibition of neutrophil extracellular trap formation and so on.^[^ ^36^, ^37^, ^38^ ^]^ In the present study, it was elucidated that miR‐16‐5p alleviated hepatic IRI through binding to the 3′UTR region of SLC39A14 mRNA, which has already identified as a key pro‐ferroptosis target above. This finding provides a new interpretation for the application of hBMSC in the therapy of hepatic IRI.

There are some limitations and perspectives in this study: (1) Although we have confirmed the role of SLC39A14 in mediating hepatocyte ferroptosis in liver graft injury, the underlying mechanisms of SLC39A14 protein regulation, including post‐transcriptional regulation or post‐translational modification in liver graft, require further investigation. (2) Given the growing potential of engineered MSCs and EVs emerging as advanced tools for biomedical applications,^[^ ^39^, ^40^ ^]^ additional research on different delivery strategies targeting SLC39A14 to alleviate hepatic IRI is expected, such as engineered MSCs‐loaded miR‐16‐5p, EVs‐loaded siRNA, et al.

In conclusion, this study revealed that SLC39A14 mediates NTBI uptake into hepatocytes, thereby triggering cell ferroptosis and exacerbating IRI in liver graft. Moreover, hBMSCs treatment reduces SLC39A14 expression level and protects hepatocytes from ferroptosis via exosomal miR‐16‐5p. These findings highlight SLC39A14 as a potential therapeutic target, and adds more evidence for the application of MSC‐based therapy for IRI in liver graft.

## 4 Experimental Section

### Ethics Statement

The study that involved human tissue samples was approved by the Research Ethics Committee of the Third Affiliated Hospital of Sun Yat‐sen University (Guangzhou, China; Approval NO. RG2023‐044‐01). Informed written consent was obtained from all patients. The animal experiments were approved by the Institutional Animal Care and Use Committee of the South China Agricultural University (Approval NO. 2022F101), and all protocols in this investigation were consistent with the ethical guidelines of the 1975 Declaration of Helsinki.

### Collection of Clinical Samples

The transplanted liver tissues were collected 2 h after reperfusion from patients who underwent their first orthotopic liver transplantation (LT) at the Third Affiliated Hospital of Sun Yat‐sen University and the healthy liver tissues adjacent to the hemangiomas were utilized for comparative analysis, which were obtained from patients who accepted hepatic hemangiomas resection. Blood samples were collected simultaneously with the tissue samples. All the tissues and blood samples were stored at ‐80 °C. The demographic characteristics of patients who underwent LT and hepatic hemangioma resection are presented in Tables S1 and S2 (Supporting Information).

### Animal Experiment

Sprague‐Dawley male rats (weight: 250–300 g) and C57BL/6 male mice (for in vivo bioluminescent imaging assay) were purchased from Sijiajingda BioTech Co., Ltd. (Guangzhou, China). All rats were raised in a temperature‐controlled barrier environment at the South China Agricultural University under a 12‐hour light‐dark cycle and had access to normal chow diet and sterile water.

To specifically knockdown *SLC39A14* expression in hepatocytes in vivo, rats weighted 150 g were administered a dose of 1.3  ×  10^13^ vg kg^−1^ adeno‐associated virus (AAV; Genechem, Shanghai, China) via tail vein 3 weeks before modeling. The virus was diluted with normal saline (NS) to a final volume of 200 µL. The AAV vector was loaded in a micro syringe attached to a 29‐gauge needle. For injection, the animals were anesthetized under isoflurane. The transfection efficiency was evaluated by fluorescence imaging and Western blotting after 3 weeks.

Rats were injected with 10 mg kg^−1^ Ferrostain‐1 (Fer‐1; Aladdin, Shanghai, China) through tail vein 24 h before autologous orthotopic liver transplantation (AOLT), or with 10^6^ MSCs through portal vein (PV) 24 hours before AOLT, or with 1 mg kg^−1^ exosomes through PV 24 h before AOLT.

### Cell Culture and Treatment

Buffalo Rat Liver‐3A (BRL3A) cells were obtained from FUSHENBIO Co., Ltd. (Shanghai, China), and were then cultured in a Dulbecco's modified Eagle's medium (DMEM) supplemented with 10% fetal bovine serum and 1% penicillin and streptomycin. As cell confluence reached 90–100%, BRL3A cells were passaged with trypsin/EDTA. Human bone marrow‐derived MSCs (hBMSCs) were obtained from Cyagen, Jiangsu, China, and cultured in a OriCell complete culture medium containing with 10% FBS and culture supplement (Cyagen). Thereafter, hBMSCs were passaged with an Animal Component‐Free Cell Dissociation Kit ((STEMCELL TECHNOLOGIES) after reaching the confluence. Notably, hBMSCs of passages 3–8 were utilized in this study. Cells were maintained in a sterile and humidified incubator with 5% CO2 at 37 °C. For the generation of *SLC39A14* stable overexpression in hepatocytes, BRL3A cells were seeded onto 6‐well plates with 40–50% confluence. Cells were then transduced with a lentivirus carrying the *SLC39A14* overexpressed vector (IGE Biotechnology). And cells transduced with lentivirus carrying pCDH‐3xFLAG vector (IGE Biotechnology) were as negative control. After 72 h of transduction, cells were subjected to puromycin selection (4 µg mL^−1^).

To establish a hypoxia/reoxygenation with ferric ammonium citrate (HR+FAC) cell model, BRL3A cells were cultured in a Roswell Park Memorial Institute (RPMI)‐1640 medium with 1 mM FAC and without FBS, glucose, and sodium pyruvate, and placed in a Galaxy 48R hypoxia incubator (Eppendorf GmbH, Hamburg, Germany) with 5% CO_2_ and 1% O_2_ at 37 °C for 12 h. Subsequently, the cells were placed in the normal cell incubator for 2 h.

BRL3A cells were pretreated with Fer‐1 (1 µM, Aladdin) for 24 h before HR. BRL3A cells or hBMSCs were transfected with miR‐16‐5p mimic (50 nmol L^−1^, RIBOBIO), or miR‐16‐5p inhibitor (100 nmol L^−1^, RIBOBIO), or si‐SLC39A14 (100 nmol L^−1^, RIBOBIO), or their corresponding negative controls (RIBOBIO). The transfection was carried out using a transfection kit (RIBOBIO) following the manufacturer's instruction, and the transfection efficiency of BRL3A cells and hBMSCs was detected by the transfection indicator kit (RIBOBIO).

### Single Cell RNA Sequencing Library Construction and Sequencing

Fresh transplanted liver tissues were stored in GEXSCOPETM tissue preservation solution (Singleron Bio Com, Nanjing, China). The specimens were washed with Hanks Balanced Salt Solution (HBSS; Servicebio, Wuhan, China) three times and cut into 1–2 mm pieces. Then, the tissue pieces were digested with 2 mL of GEXSCOPETM tissue dissociation solution (Singleron) at 37 °C for 15 min with oscillation. Next, the samples were filtered through 40‐µm sterile strainers and centrifuged at 800 × g for 5 min. After centrifugation, the supernatants were discarded and the cell precipitate was suspended in 1 mL phosphate‐buffered saline (PBS; KeyGEN Bio Tech, Jiangsu, China). Then, cells were incubated in GEXSCOPETM red blood cell lysis buffer (Singleron) at 25 °C for 10 min to remove red blood cells. Subsequently, the solution was centrifuged at 500 × g for 5 min and resuspended in PBS (Concentration: 1 × 10^5^ cells mL^−1^).

Single cells were loaded onto a microfluidic chip and the scRNA‐seq libraries were constructed using the GEXSCOPE Single‐Cell RNA Library Kit (Singleron Biotechnologies) following the Singleron GEXSCOPE operation manual. The libraries were diluted to a concentration of 4 nM and sequenced by the Illumina Novaseq6000 sequencing platform with 150 bp paired‐end reads.

### Quality Control and Single Cell RNA Sequencing Data Pre‐Processing

Raw reads were processed to generate gene expression profiles using the standard internal pipeline from Singleron. In brief, after filtering read 1 without poly T tails, cell barcode and unique molecular identifiers (UMIs) were extracted. Then, adapters and poly A tails were trimmed (fastp V1) before aligning the read 2 to the reference genome in Ensemble database (STAR 2.5.3a and featureCounts 1.6.2). Reads with the same cell barcode, UMIs and genes were grouped together to calculate the number of UMIs per gene per cell. The UMI count tables of each cellular barcode were used for further analysis.

The raw output data were processed with the Seurat package (http://satijalab.org/seurat/, R package, v.3.0.1) in R software (version 3.6.1) for cell types recognition and clustering analysis. The expression matrix was imported into R software using the read.table function, and cell clustering analysis was processed using the FindCluster function (parameter resolution 0.6). The differentially expressed genes (DEGs) between different samples or continuous clusters were identified using the findmarker function. Biological functions or pathways significantly associated with specific expressed genes were identified by GO functional enrichment analysis on the gene set using clusterProfiler software.

### Data Integration, Dimension Reduction, and Cell Clustering

The Seurat object containing gene expression data from individual samples was processed using the Read10× function. In this process, the gene expression values for each sample were represented as the fraction of the gene and multiplied by 10 000. These values were then converted into their natural logarithm and normalized by adding 1 to avoid taking the logarithm of 0. Next, we identified the top 3000 highly variable genes (HVGs) from the normalized expression matrix. These HVGs were centered and scaled before performing the principal component analysis (PCA). To address any potential batch effects, the Harmony package (version 1.0) of R was utilized based on the top 50 PCA components identified.

### Annotating Cell Clusters

The clustering analysis was performed using the integrated joint embedding generated by the Harmony algorithm. This was combined with the Louvain algorithm to compute a shared nearest‐neighbor graph. The FindClusters function from the Seurat package was used to implement the Louvain algorithm for this purpose. The identified clusters were visualized on the 2D map produced with the UMAP method. To annotate the cell clusters, DEGs that exhibited high discrimination abilities between the groups were identified with the FindAllMarkers function in Seurat using the default non‐parametric Wilcoxon rank sum test with Bonferroni correction. The cell groups were annotated based on the identified DEGs as well as the well‐known cellular markers from the literatures.

### Gene Set Enrichment Analysis (GSEA)

Gene Set Enrichment Analysis (GSEA) was also conducted with curated gene sets to identify the pathways that were upregulated or downregulated between the different cell clusters. For this analysis, a modified version of the competitive gene set enrichment test CAMERA was utilized, which has been implemented in the SingleSeqGset (version 0.1.2) R package. To perform the analysis, we first calculated the mean gene expression level and then used the log2 fold change (FC) between the specific cell cluster and the other cells as the test statistic. The 50 hallmark gene sets were utilized from the MSigDB databases (https://www.gsea‐msigdb.org/gsea/msigdb) for the GSEA analysis. These gene sets represent well‐defined biological pathways and processes.

### Autologous Orthotopic Liver Transplantation (AOLT) Rat Model

AOLT rat model was established according to the standard procedure reported previously.^[^ ^25^ ^]^ Briefly, the rats were under deep anesthesia with isoflurane and were heparinized systemically with 50 U heparin in 1 mL NS via tail vein injection. The abdomen was opened and the liver falciform ligament was resected first. Then, left superior phrenic vein was ligated with 6‐0 absorbable suture, and a 0 suture was put under the suprahepatic vena cava (SVC) for the later blockage. Splenogastric vein and right suprarenal vein were respectively separated and ligated with 4‐0 absorbable sutures. After the portal vein (PV) and infrahepatic vena cava (IVC) were both liberated, they were clipped with microvascular clamps. The PV was punctured with a trocar needle, through which 1.5 mL NS with 25 U mL^−1^ heparin was slowly injected to expel the blood from hepatic sinus to right atrium. Till the liver turned yellow, the SVC was ligated with 0 suture. Then, 20 mL 4 °C NS with 12.5 U mL^−1^ heparin was continuously pumped from the PV and meanwhile a 1 mm incision was made on the IVC wall as an outflow tract. The duration of the anhepatic phase was ≈20 ±  1 min. In the end of the anhepatic phase, the openings of PV and SVC were stitched with 9‐0 absorbable suture. Following the ischemia stage, the microvascular clamps and the ligation were all removed, and the rats were rewarmed by peritoneal lavage with preheated NS with 0.002 mg mL^−1^ epinephrine and 0.004 mg mL^−1^ norepinephrine. Finally, compound lidocaine cream (Ziguang Pharmaceutical Co. Ltd, Beijing China) was applied to the incision surface.

### Histological Analysis

The liver samples were fixed in 10% (v/v) buffered formalin (Nanjing Keygen Biotech, Jiangsu, China) overnight on a shaker at room temperature, embedded in paraffin, and cut into 5‐µm‐thick sections. Liver sections were stained with hematoxylin and eosin (H&E), and Suzuki's score was used as a standard to evaluate the pathological damage.

### Quantitative Real‐Time Polymerase Chain Reaction (RT‐qPCR)

Total RNA was extracted by RNA extraction solution (Servicebio) and quantified by ultramicro spectrophotometer (ThermoFisher Scientific). For mRNA detection, total RNA was reverse‐transcribed using HiScript III RT SuperMix for qPCR (Vazyme, Nanjing, China); real‐time PCR amplification was carried out by ChamQ Universal SYBR qPCR (Vazyme). β‐actin was used as the internal control, and relative mRNA expression was calculated using the ΔΔCt method. The following gene‐specific primers were used for RT‐qPCR: β‐actin, F‐AAATCGTGCGTGACATCAAAGA and R‐GCCATCTCCTGCTCGAAGTC; SLC39A14, F‐gagcaccatcacggacat and R‐ccctttgagccagtagca. For miRNA detection, reverse transcription of total RNA and real‐time PCR amplification were carried out using Bulge‐Loop miRNA qRT‐PCR Starter Kit (RiboBio, Guangzhou, China). The primer set of U6 (#MQPS0000002, RiboBio), has‐miR‐16‐5p (#MQPS0000695, RiboBio), has‐miR‐100‐5p (#MQPS0000417, RiboBio) and has‐miR‐34a‐5p (#MQPS0001096, RiboBio) were used in this experiment.

### Immunofluorescence and Immunohistochemical Staining

The experiments were carried out following the previously reported procedure.^[^ ^25^ ^]^ After dewaxing, antigen retrieval and permeabilization as well as being blocked with 5% bovine serum albumin (BSA) or 5% goat serum, the paraffin slides were incubated overnight at 4 °C with primary antibodies against SLC39A14 (1:100, #DF14224, Affinity), albumin (1:100, #66 051, Proteintech), GPX4 (1:100, #DF6701, Affinity), and 4‐HNE (1:100, # ab48506, abcam). For immunofluorescence staining, the slides were then incubated with Alexa Fluor 488‐conjugated (#4412S, Cell Signaling Technology, MA, USA) or Alexa Fluor 594‐conjugated (#ab150080, abcam) secondary antibody in dark for 1 h at room temperature. The slides were imaged with a fluorescence microscope (ThermoFisher Scientific). For immunohistochemical staining, 3% (v/v) H2O2 was used to clear the endogenous peroxidase activity and a horseradish peroxidase (HRP) conjugated Goat Anti‐Rabbit IgG (H+L) (#GB23303, Servicebio) was used as the secondary antibody. Then, the slides were stained with DAB Chromogenic Kit (#G1212, Servicebio). The slides were imaged with a pathological section scanner (3DHISTECH).

### Western Blotting

The procedures for total protein extraction and western blotting were performed as described in the previous study.^[^ ^41^ ^]^ The following antibodies were used in this study: SLC39A14 (1:1000, #DF14224, Affinity), GPX4 (1:1000, #DF6701, Affinity), FTH‐1 (1:1000, # ab75972, abcam), GAPDH (1:5000, #2118, CST), β‐actin (1:5000, #4970, CST), HRP‐conjugated Anti‐rabbit IgG (1:20 000, #7074, CST) and HRP‐conjugated Anti‐mouse IgG (1:20 000, #7076, CST).

### Detection of Serum Non‐Transferrin Bound Iron (NTBI) and Tissue Ferrous Iron (Fe^2+^)

Detection of serum non‐transferrin bound iron was processed as previously described.^[^ ^42^ ^]^ Briefly, 450 µL fresh serum was mixed with 50 µL 800 mM Nitrilotriacetic acid (NTA) solution to separate the NTA‐Fe complex. Then, the mixed solution was transferred to Centricon‐30 filter column (Amicon Ultra) and was centrifuged at 3000 g at 4 °C for 1 h. After centrifugation, 100 µL of filtrate was collected into a new centrifuge tube, which was added with 100 µL of MOPs buffer, 25 µL of BPT solution and 25 µL of TGA solution. The mixture was incubated at room temperature for 1 h before measuring the absorbance of each well using a microplate reader at 537 nm, and the NTBI concentration was calculated based on the standard series. Tissue ferrous iron level was detected using the Ferrous Ion Content Assay Kit (Biosharp).

### Malondialdehyde (MDA) Detection

MDA levels, which are representative of lipid peroxidation, were detected by the MDA assay kit (Nanjing Jiancheng Bioengineering Institute) following the manufacture instruction.

### Transmission Electron Microscopy (TEM)

The morphology of mitochondria in liver tissues and of exosomes were examined by TEM. The fresh liver tissues were fixed with malondialdehyde and stored at 4 °C. The rest of procedures were performed by Servicebio and the grids were visualized with Hitachi TEM system at 80.0 kV.

### Transwell Assay

24‐well transwells inserted with 0.4 µm pore‐sized filters (Corning, NY, USA) were used for the co‐culture system in this assay. hBMSCs were plated into the upper chamber (1 × 10^5^ cells per well) and were cultured for 24 h. Then, the upper chambers were moved to the 24‐wells plated with BRL3A. After BRL3A cells were co‐cultured with hBMSCs for 24 hours, the upper chambers were removed and the cells were used for hypoxia and reoxygenation (HR) experiment.

### Isolation of Exosomes from hBMSCs

The MISEV criteria were followed to isolate and identify exosomes derived from hBMSCs (MSC‐exo).^[^ ^43^ ^]^ Briefly, hBMSCs of passage 3–8, used for exosome isolation, were cultured with serum‐free MesenCultTM‐ACF Plus Medium (STEMCELL TECHNOLOGIES). The conditioned medium was collected and filtered by 0.22 µm PES membrane filer unit (Merck Millipore, Darmstadt, German) to remove cells. Then, the filtrate was centrifuged 10 000 g for 30 min to remove cell debris, followed by a 100 000‐NMWL cutoff membrane concentration (Merck Millipore). The ultrafiltrate was then ultra‐centrifuged at 100 000 g for 70 min at 4 °C using the SW 32Ti Rotor by BECKMAN OPTIMAL‐90K (Beckman Coulter, California, USA). The pellets were resuspended with PBS and ultra‐centrifuged again using the SW 32Ti Rotor at 100 000 g for 70 min at 4 °C. The final pellets were resuspended with PBS and stored at −80 °C.

### Nanoparticle Tracking Analysis

The size distribution of MSC‐exo was evaluated by the Flow NanoAnalyzer (NanoFCM, Xiamen, China). 5 µL MSC‐exo obtained from ultra‐centrifuge was diluted to 30 µL with PBS. The instrument performance was tested with the standard before the measurement of MSC‐exo. Three different samples were tested in this assay.

### In Vivo Bioluminescent Imaging Assay

Mice for this assay were anesthetized and injected with exosomes that were labeled with PKH26 (MedChemExpress) through tail vein. Mice were placed on IVIS Lumina III In Vivo Imaging System on ventrodorsal position at 6, 12, and 24 h after injection.

### Exosomes Internalization Assay

MSC‐exo was labeled with 1 µM CM‐Dil (Invitrogen) for 30 min and PBS added with Dil was as control. The labeled MSC‐exo and PBS was then respectively added to BRL3A for co‐culture for 24 h. BRL3A was washed with PBS three times and the cytoskeleton was stained with FITC‐phalloidin (Servicebio) according to the manufacturer's instruction. The images were captured with a fluorescence microscope (Thermo Fisher Scientific).

### Intracellular Fe^2+^ and Lipid Peroxidation (LPO) Detection

The levels of intracellular Fe^2+^ and LPO were detected respectively by FerroOrange probe (DOJINDO, Shanghai, China) and liperfluo probe (DOJINDO) according to the manufacturer. Briefly, for FerroOrange staining, after washed with PBS, BRL3A were trypsinized and then resuspended with 1 µM FerroOrange working solution 37 °C for 30 min followed by the flow cytometry. For liperfluo staining, BRL3A was washed with PBS and then trypsinized. The pellets were incubated with 1 µM Liperfluo working solution diluted in non‐serum MEM medium at 37 °C for 30 min. After washed with PBS twice, the cells were resuspended with HBSS. The fluorescence intensity was measured by flow cytometry (FerroOrange: Ex/Em = 561/570‐620 nm, Liperfluo: Ex/Em = 488/500–550 nm).

### Dual Luciferase Reporter Assay

Wild‐type (WT) or mutated (MUT) sequences corresponding to the 3′‐UTR of SLC39A14 mRNA were synthesized by IGE Biotechnology (Guangzhou, China) and cloned into the pmirGLO dual‐Luciferase miRNA target expression vector (Promega, Madison, USA). The constructed plasmids together with miR‐16‐5p mimic were transfected into human embryonic kidney 293T Cells (HEK293T) using ExFect Transfection Reagent (Vazyme) and a transfection kit (RIBOBIO). Luciferase activity was measured by dual‐luciferase reporter assay system (Promega). Renilla luciferase activity was used as the internal reference.

### Cell Counting Kit 8 (CCK8) Assay

CCK8 was used to measure cell survival rate as reported previously.^[^ ^25^ ^]^ Briefly, 10 µL CCK8 solution was added in normal BRL3A or BRL3A after hypoxia which were seeded in 96‐wells plate and cultured in 100 µL corresponding cultural medium. Then, the plates were continuously incubated at 37 °C for 2 h in a cell incubator. The absorbance at 450 nm was measured by a microplate reader (Epoch Technologies).

### miRNA Expression Profiling

A total of three MSC‐exo samples were sequenced on DNBSEQ platform, with an average yield of 33.6 M reads per sample. The average alignment ratio of the sample comparison genome was 24.36%. A total of 493 miRNAs were detected.

### Statistical Analysis

Statistical analysis was conducted using GraphPad Prism software (version 9.0). Quantitative data represents at least three independent experiments. For the comparation of mean values between two groups, unpaired two‐tailed student's *t* test was used. One‐way analysis of variance (ANOVA) followed by Tukey's post‐hoc tests were used to assess the statistical significance of the mean values of more than two groups. A two‐tailed *P* value less than 0.05 was considered statistically significant difference.

## Conflict of Interest

The authors declare no conflict of interest.

## Author Contributions

Z.D., W.Z., Y.G., Z.Y., and X.L. contributed equally to this work. D.Y., Z.H., and G.L. designed the projects and supervised this study. Z.D., W.Z., Y.G., Z.Y., and X.L. performed most of the experiments and analyzed the data. X.L., G.S., and E.X. participated in experimental work and data analysis. Z.D., D.Y., and Z.H. drafted and edited the manuscript. All authors have read and approved the article.

## Supporting information



Supporting Information

## Data Availability

The data that support the findings of this study are available from the corresponding author upon reasonable request.
