# Supplementary material for: Mesenchymal Stem Cells Prevent SLC39A14‐Dependent Hepatocyte Ferroptosis through Exosomal miR‐16‐5p in Liver Graft
Source: Adv Sci (Weinh). 2024 Dec 16;12(6):2411380. doi: 10.1002/advs.202411380 (PMC11809355; doi:10.1002/advs.202411380)
Supplement: Supplementary file 1 — Supporting Information [file ADVS-12-2411380-s001.docx]

**Mesenchymal stem cells prevent SLC39A14-dependent hepatocyte ferroptosis through exosomal miR-16-5p in liver graft**

Zhizhao Deng#, Weiqi Zeng#, Yingxin Gao#, Zhenyu Yang#, Xinling Luo#, Xianlong Li, Guoliang Sun, Erfeng Xiong, Fei Huang, Gangjian Luo*, Ziqing Hei* and Dongdong Yuan*

**Content**

[Supplementary Figure S1 2](#_Toc184162645)

[Supplementary Figure S2 4](#_Toc184162646)

[Supplementary Figure S3 5](#_Toc184162647)

[Supplementary Figure S4 6](#_Toc184162648)

[Supplementary Figure S5 7](#_Toc184162649)

[Supplementary Figure S6 8](#_Toc184162650)

[Supplementary Figure S7 9](#_Toc184162651)

[Supplementary Figure S8 10](#_Toc184162652)

[Supplementary Figure S9 11](#_Toc184162653)

[Supplementary Figure S10 12](#_Toc184162654)

[Supplementary Figure S11 12](#_Toc184162655)

[Supplementary Figure S12 13](#_Toc184162656)

[Supplementary Table S1 14](#_Toc184162657)

[Supplementary Table S2 15](#_Toc184162658)

# Supplementary Figure S1

**
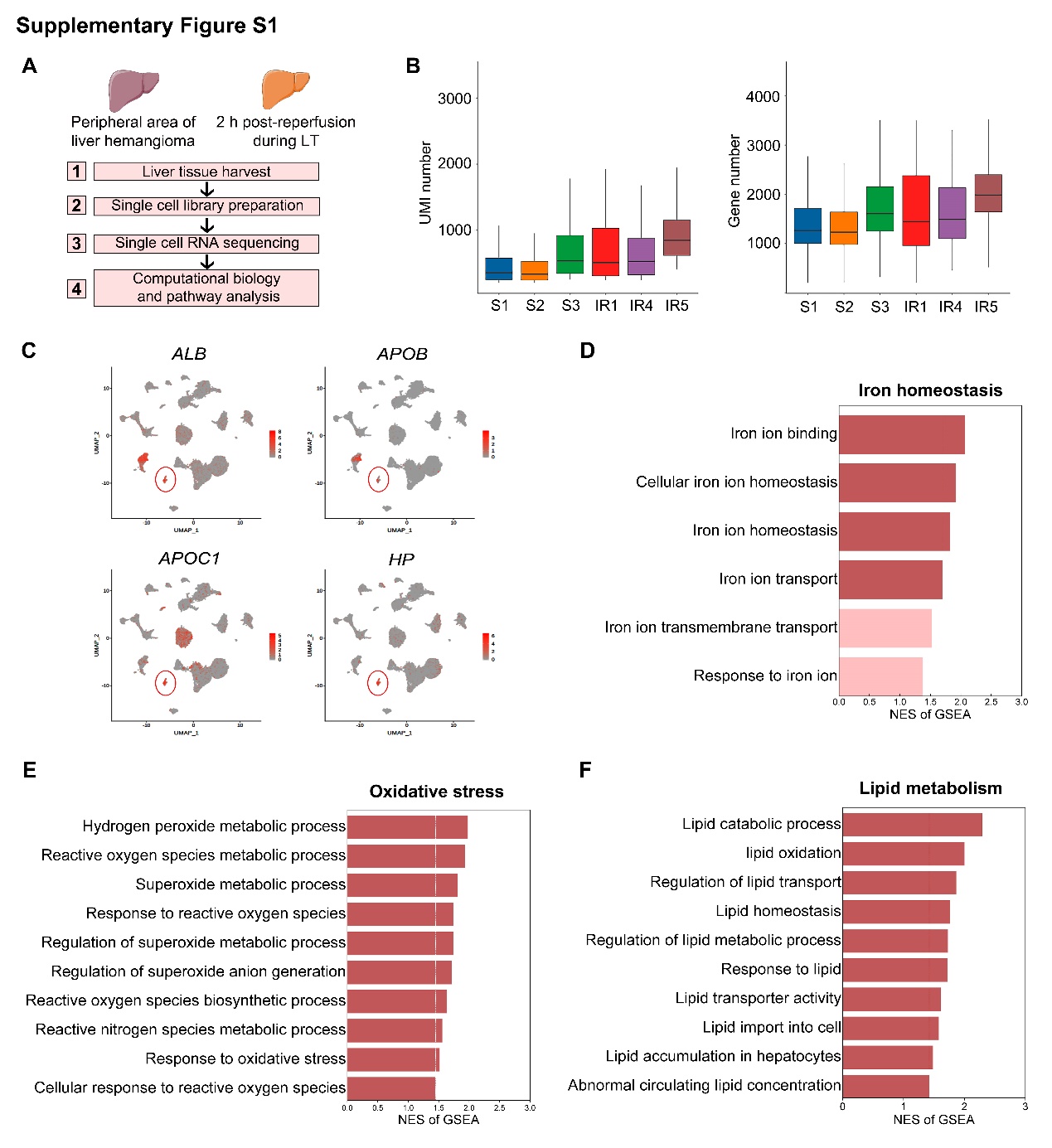
**

**Supplementary Fig. S1 scRNA-seq reveals hepatocyte characterized with ferroptosis in liver graft. (A)** Schematic workflow of the experimental strategy. **(B)** The box plot revealing the distribution of detected total UMIs and gene numbers per cell of the single cells in each liver samples from patients receiving hepatic hemangiomas resection and liver transplantation. Each box indicates the interquartile range (between the 25th and 75th percentile) with mid-point data. The whiskers represent the lower and upper value within 1.5 times the interquartile range. **(C)** Bubble chart showing the top 5 of different expressed genes among each cell clusters. **(D)** UMAP plot showing the distribution of the hepatocyte marker (ALB, APOB, APOC1 and HP) by scRNA-seq analysis. **(E-G)** The bar plot of GSEA of the hallmark gene sets in MSigDB database revealing the enrichment of iron homeostasis (E), oxidative stress (F) and lipid metabolism (G) related Gene Ontology (GO) terms in hepatocyte cluster by scRNA-seq analysis.

# Supplementary Figure S2


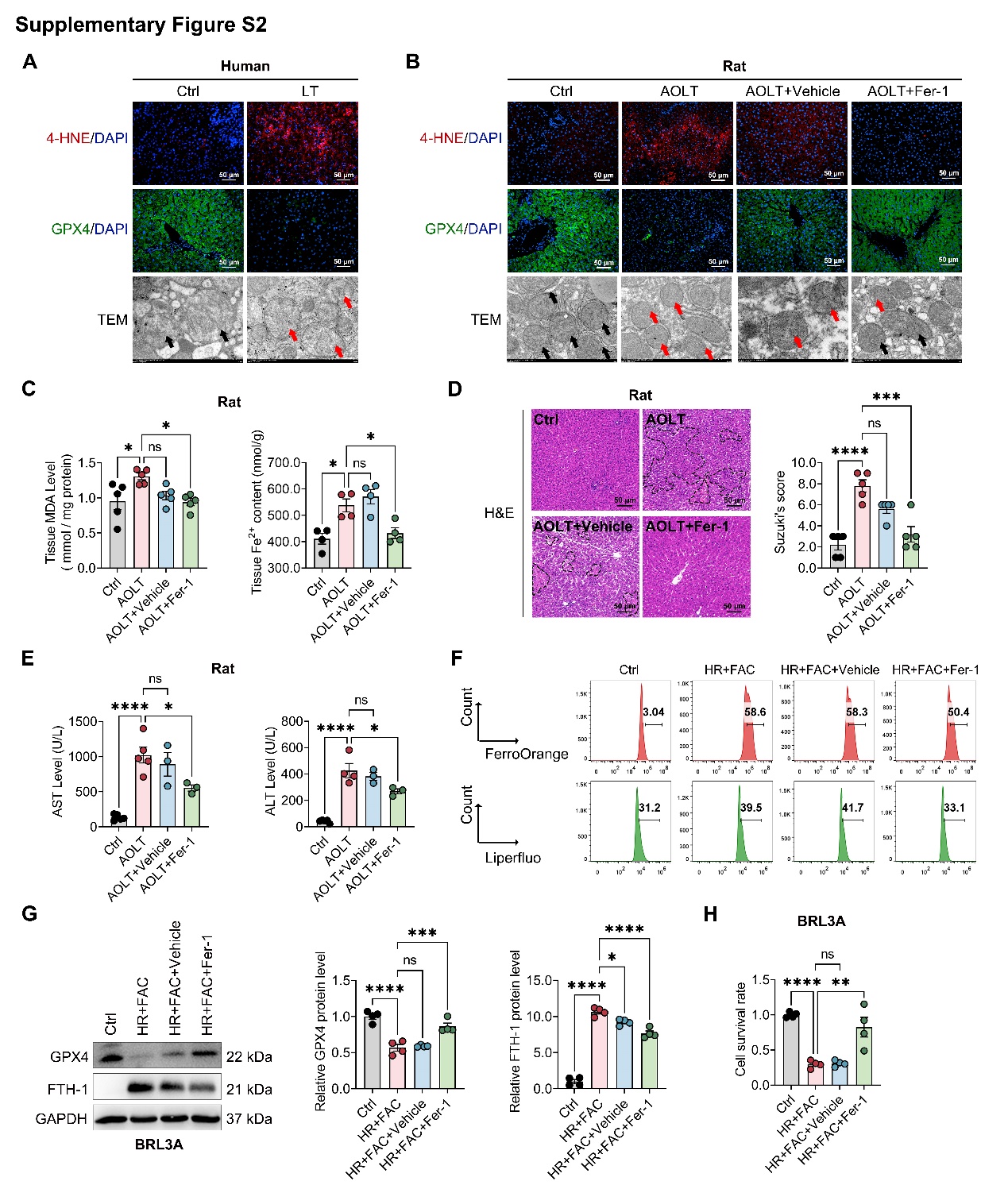


**Supplementary Fig. S2 Inhibiting hepatocyte ferroptosis with Fer-1 reduces hepatic ischemia and reperfusion injury. (A, B)** Immunofluorescence staining showing the expression levels of 4-HNE and GPX4 protein and transmission electron microscopy (TEM) showing the mitochondrial morphology of hepatocytes in liver tissues from patients and rats. **(C)** Level of MDA and iron in liver tissues from rats. Data are shown as mean ± SD, n = 5. **(D and E)** Evaluation of liver graft injury. (D) Pathological analysis by H&E staining and Suzuki’s score. Data are shown as mean ± SD, *n* = 5. (E) Serum AST and ALT concentration. Data are shown as mean ± SD, *n* = 3-5. **(F)** Flow cytometry analysis of ferrous iron level by ferroOrange probe and lipid peroxidation level by liperfluo probe in BRL3A cells. **(G)** Representative images of western blotting and relative quantitative analysis showing GPX4 and FTH-1 protein level in BRL3A cells. **(H)** CCK8 assessed cell damage severity in BRL3A cells. Data are shown as mean ± SEM, n = 4. *: *p* < 0.05, **: *p* < 0.01, ***: *p* < 0.001, ****: *p* < 0.0001. Ctrl, control; LT, liver transplantation; AOLT, autologous orthotopic liver transplantation; HR+FAC, hypoxia and reperfusion with ferric ammonium citrate; Fer-1, ferrostain-1.

# Supplementary Figure S3


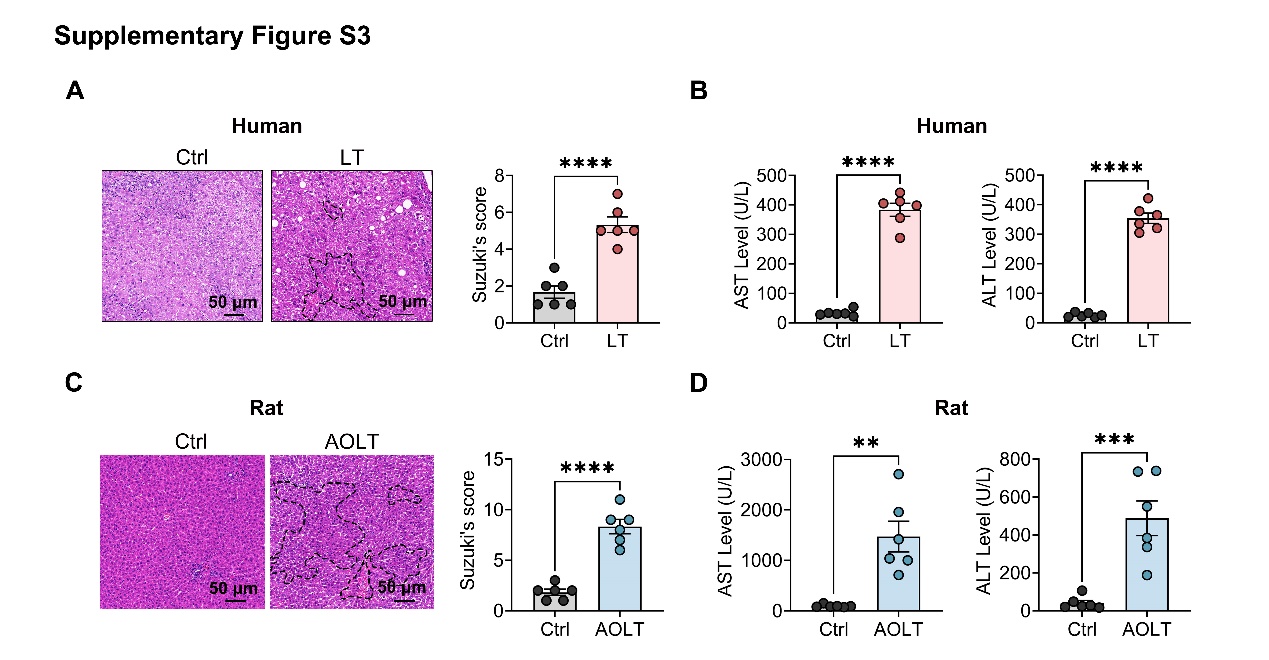


**Supplementary Fig. S3 Evaluation of hepatic ischemia and reperfusion injury in liver grafts from human and rats. (A)** Pathological analysis by H&E staining and Suzuki’s score on human liver tissues. Data are shown as mean ± SD, n = 6. **(B)** Serum AST and ALT concentration. Data are shown as mean ± SD, n = 6. **(C)** Pathological analysis by H&E staining and Suzuki’s score on liver tissues from rats. Data are shown as mean ± SD, n = 6. **(D)** Serum AST and ALT concentration. Data are shown as mean ± SD, n = 6. Ctrl, control; LT, liver transplantation; AOLT, autologous orthotopic liver transplantation.

# Supplementary Figure S4


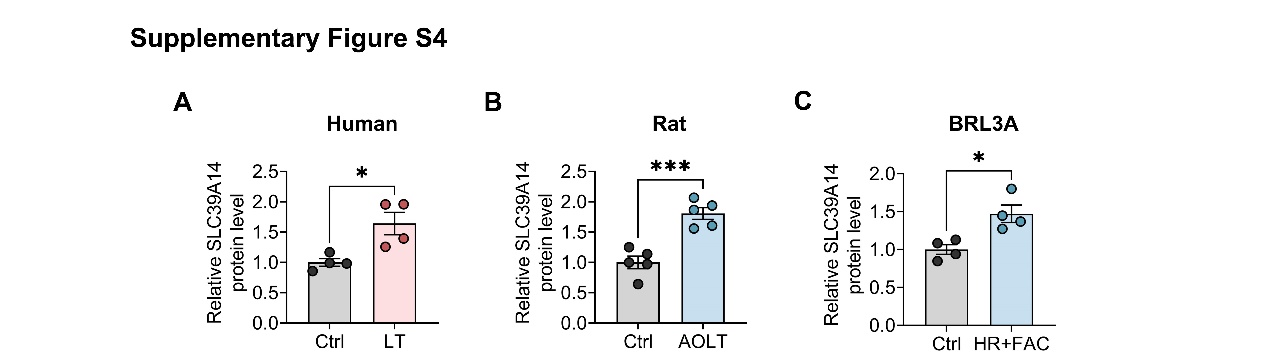


**Supplementary Fig. S4 Relative SLC39A14 expression in liver tissues and hepatocytes. (A)** Relative quantitative analysis of SLC39A14 protein level in human liver tissues. Data are shown as mean ± SEM, n = 4. **(B)** Relative quantitative analysis of SLC39A14 protein level in liver tissues from rats. Data are shown as mean ± SEM, n = 5. **(C)** Relative quantitative analysis of SLC39A14 protein level in BRL3A cells. Data are shown as mean ± SEM, n = 4. *: *p* < 0.05, **: *p* < 0.01, ***: *p* < 0.001, ****: *p* < 0.0001. Ctrl, control; LT, liver transplantation; AOLT, autologous orthotopic liver transplantation; HR+FAC, hypoxia and reperfusion with ferric ammonium citrate.

# Supplementary Figure S5

**
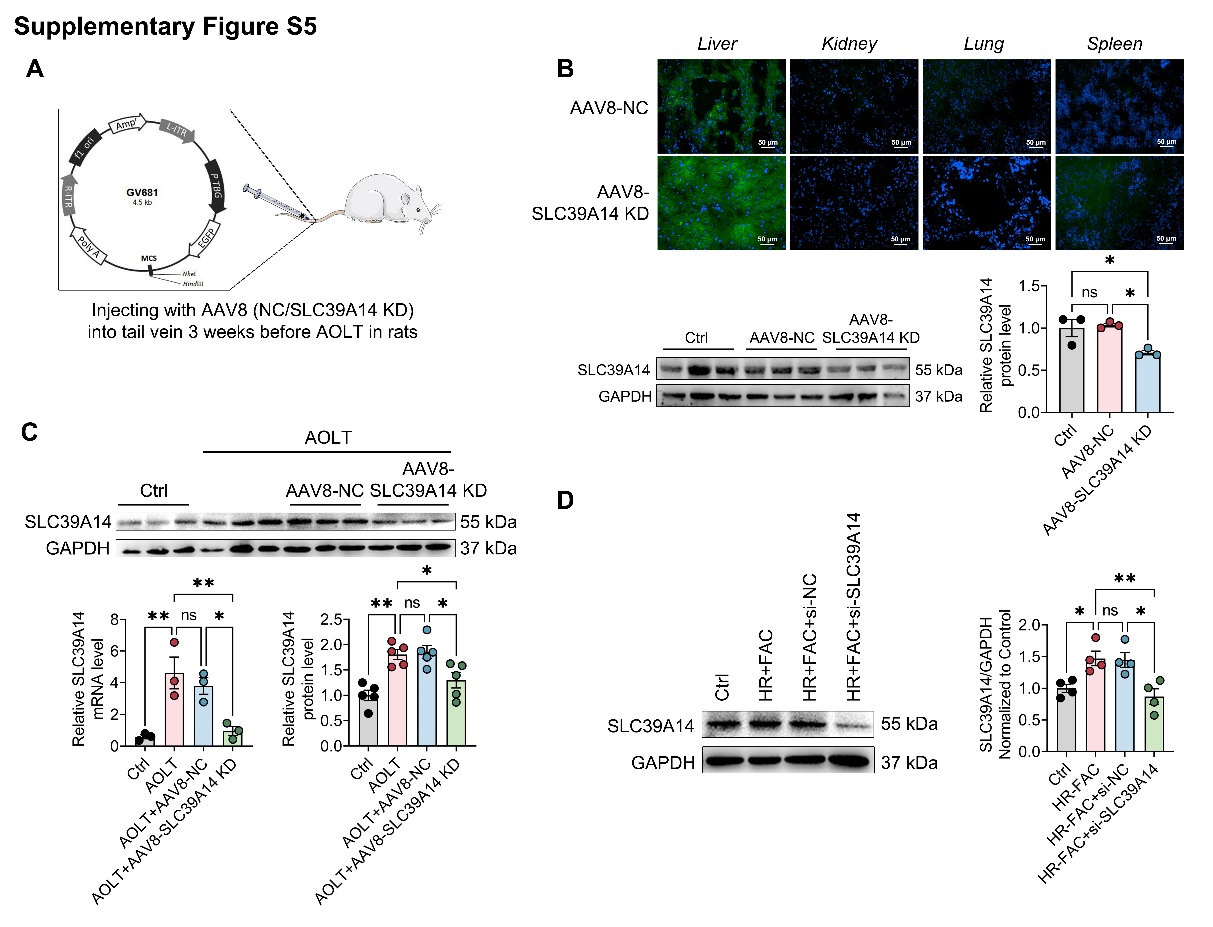
**

**Supplementary Fig. S5 SLC39A14 expression was knocked down by AAV and siRNA respectively in rats and BRL3A cells.** **(A)** Schematic map of AAV injection in rats. **(B)** Transfection effect of AAV in different organs in rats and western blotting showing the inhibitive efficiency of SLC39A14 expression in the liver in rats. Data are shown as mean ± SEM, n = 3. **(C)** RT-qPCR detecting the level of SLC39A14 mRNA, and representative images of western blotting and relative quantitative analysis showing the level of SLC39A14 protein in rats treating with/without AOLT and with/without AAV carrying shRNA-SLC39A14. Data are shown as mean ± SEM, n = 3-5. (D) Representative images of western blotting and relative quantitative analysis showing the level of SLC39A14 protein in BRL3A cells treating with/without HR+FAC and with/without siRNA-SLC39A14. Data are shown as mean ± SEM, n = 4. *: *p* < 0.05, **: *p* < 0.01, ***: *p* < 0.001, ****: *p* < 0.0001. Ctrl, control; AOLT, autologous orthotopic liver transplantation; HR+FAC, hypoxia and reperfusion with ferric ammonium citrate.

# Supplementary Figure S6


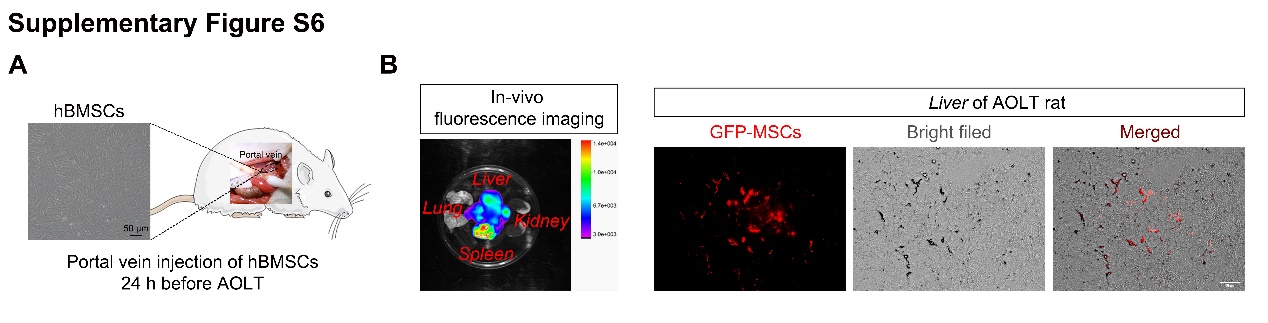


**Supplementary Fig. S6 Injection of hBMSCs in rats. (A)** Schematic map of hBMSCs injection through portal vein in rats. **(B)** *In vivo* imaging (PKH26-labled hBMSCs) and GFP-MSCs (in red fluorescence) used for tracing hBMSCs.

# Supplementary Figure S7


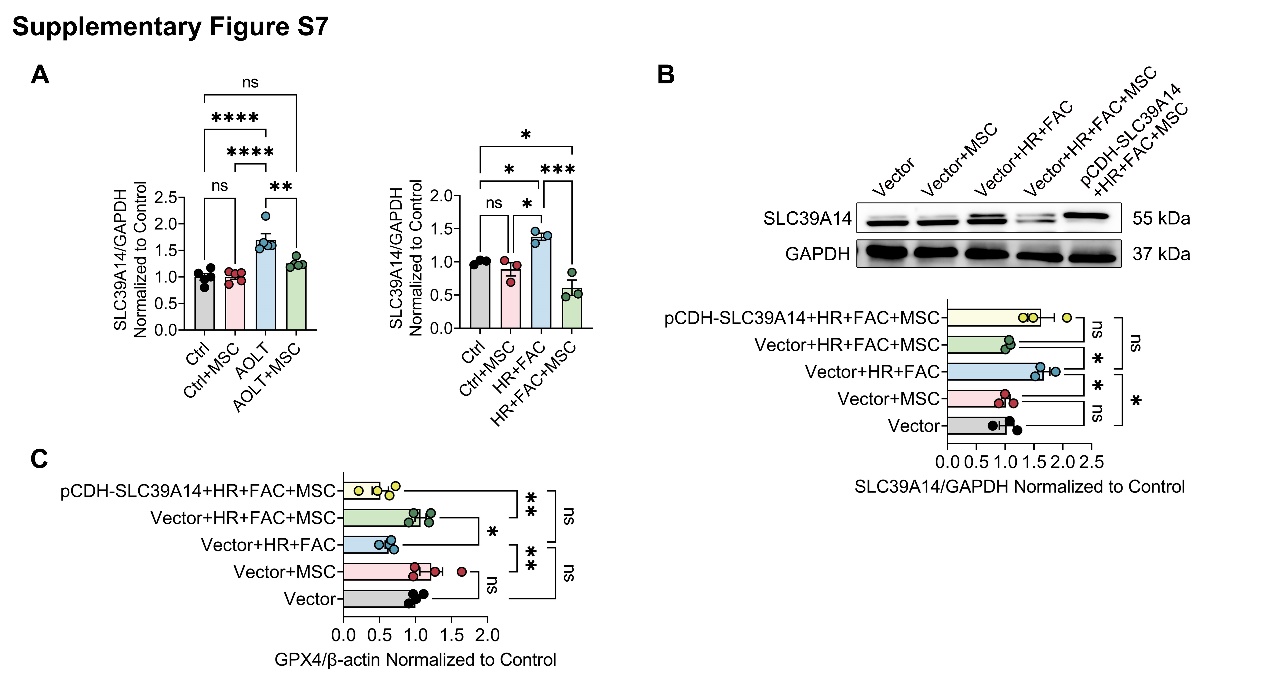


**Supplementary Fig. S7 Relative expression level of SLC39A14 and GPX4 in rats and BRL3A cells treating with hBMSCs. (A)** Relative quantitative analysis showing the level of SLC39A14 protein in AOLT rats and HR-FAC-induced BRL3A cells with/without hBMSCs. Data are shown as mean ± SEM, n = 3-5. **(B)** Representative images of western blotting and relative quantitative analysis showing the level of SLC39A14 protein in BRL3A cells treating with pCDH-NC or *SLC39A14* overexpression vector and with/without hBMSCs. Data are shown as mean ± SEM, n = 3. **(C)** Relative quantitative analysis showing the level of GXP4 protein in BRL3A cells treating with pCDH-NC or *SLC39A14* overexpression vector and with/without hBMSCs. Data are shown as mean ± SEM, n = 4. *: *p* < 0.05, **: *p* < 0.01, ***: *p* < 0.001, ****: *p* < 0.0001. Ctrl, control; AOLT, autologous orthotopic liver transplantation; HR+FAC, hypoxia and reperfusion with ferric ammonium citrate.

# Supplementary Figure S8


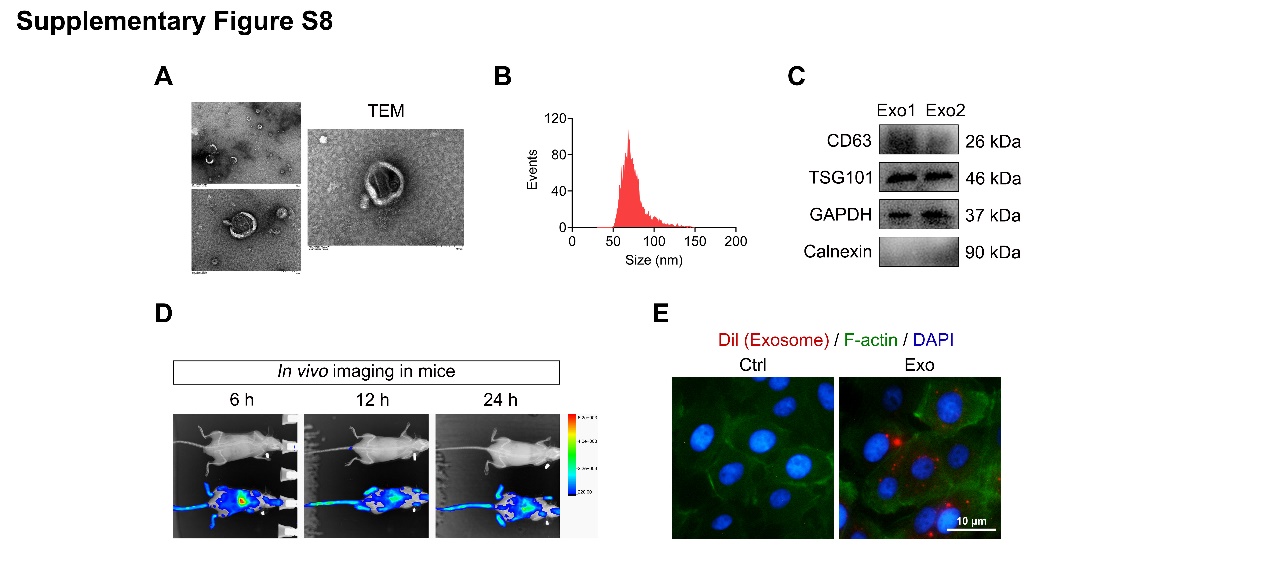


**Supplementary Fig. S8 Characteristic of exosomes derived from hBMSCs. (A)** Transmission electron microscope detecting the morphology of exosomes derived from hBMSCs. **(B)** Nanoparticle tracking analysis detecting the size of exosomes derived from hBMSCs. **(C)** Representative images of western blotting showing the protein markers of exosomes derived from hBMSCs. **(D)** *In vivo* imaging (PKH26-labled exosomes) used for tracing exosomes. **(E)** hBMSCs-derived exosomes labelled with Dil were taken up by BRL3A cells. The cytoskeleton in BRL3A cells was stained by FITC-phalloidin (green).

# Supplementary Figure S9


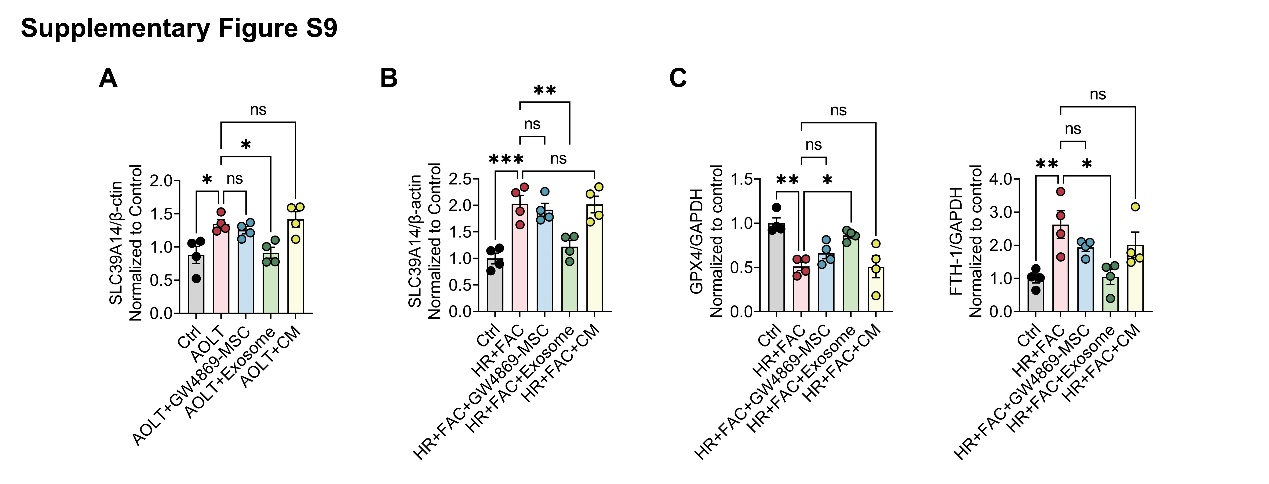


**Supplementary Fig. S9 Relative expression level of SLC39A14, GPX4 and FTH-1 in rats and BRL3A cells treating with exosomes. (A)** Relative quantitative analysis showing the level of SLC39A14 protein in normal rats and AOLT rats with GW4869-treated hBMSCs, exosomes or conditioned medium (CM). Data are shown as mean ± SEM, n = 4. **(B)** Relative quantitative analysis showing the level of SLC39A14 protein in normal and HR+FAC-induced BRL3A cells with GW4869-treated hBMSCs, exosomes or conditioned medium (CM). Data are shown as mean ± SEM, n = 4. **(C)** Relative quantitative analysis showing the level of GXP4 and FTH-1 protein in normal and HR+FAC-induced BRL3A cells with GW4869-treated hBMSCs, exosomes or conditioned medium (CM). Data are shown as mean ± SEM, n = 4. *: *p* < 0.05, **: *p* < 0.01, ***: *p* < 0.001, ****: *p* < 0.0001. Ctrl, control; AOLT, autologous orthotopic liver transplantation; HR+FAC, hypoxia and reperfusion with ferric ammonium citrate.

# Supplementary Figure S10


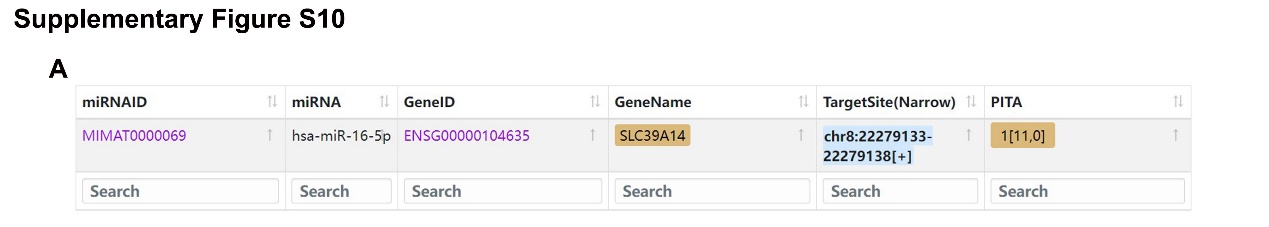


**Supplementary Fig. S10 Prediction of target site of miR-16-5p. (A)** Target site of miR-16-5p on SLC39A14 protein was predicted using starBase (ver. 2.0).

# Supplementary Figure S11


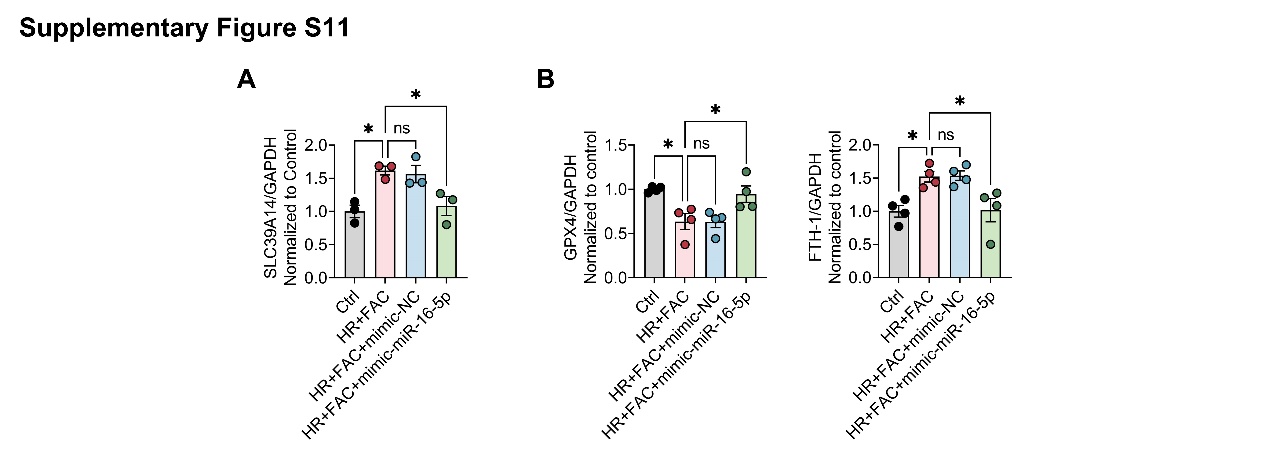


**Supplementary Fig. S11 Relative expression level of SLC39A14, GPX4 and FTH-1 in BRL3A cells treating with miR-16-5p mimic. (A)** Relative quantitative analysis showing the level of SLC39A14 protein in normal and HR+FAC-induced BRL3A cells with mimic-NC or mimic-miR-16-5p. Data are shown as mean ± SEM, n = 3. **(B)** Relative quantitative analysis showing the level of GPX4 and FTH-1 protein in normal and HR+FAC-induced BRL3A cells with mimic-NC or mimic-miR-16-5p. Data are shown as mean ± SEM, n = 4. *: *p* < 0.05, **: *p* < 0.01, ***: *p* < 0.001, ****: *p* < 0.0001. Ctrl, control; HR+FAC, hypoxia and reperfusion with ferric ammonium citrate.

# Supplementary Figure S12

**
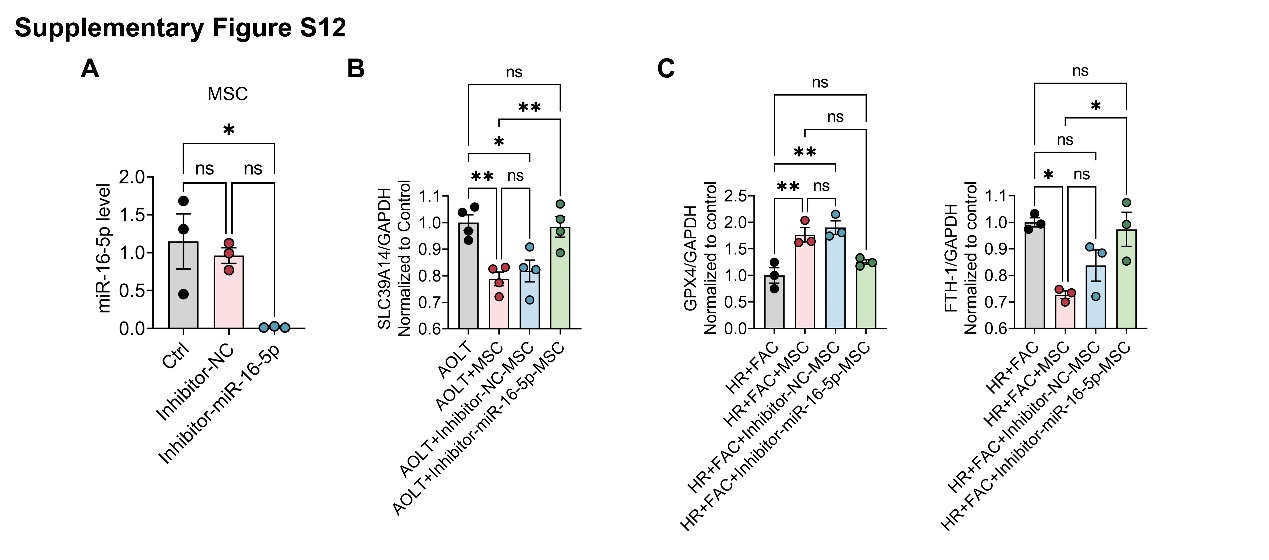
**

**Supplementary Fig. S12 Relative expression level of SLC39A14, GPX4 and FTH-1 in rats and BRL3A cells treating with hBMSCs transfected with miR-16-5p inhibitor. (A)** RT-qPCR detecting miR-16-5p level in hBMSCs transfected with miR-16-5p inhibitor. Data are shown as mean ± SD, n = 3. **(B)** Relative quantitative analysis showing the level of SLC39A14 protein in normal and HR+FAC-induced BRL3A cells co-cultured with hBMSCs that were transfected with inhibitor-NC or inhibitor -miR-16-5p. Data are shown as mean ± SEM, n = 4. **(C)** Relative quantitative analysis showing the level of GPX4 and FTH-1 protein in normal and HR+FAC-induced BRL3A cells co-cultured with hBMSCs that were transfected with inhibitor-NC or inhibitor -miR-16-5p. Data are shown as mean ± SEM, n = 3. *: *p* < 0.05, **: *p* < 0.01, ***: *p* < 0.001, ****: *p* < 0.0001. Ctrl, control; HR+FAC, hypoxia and reperfusion with ferric ammonium citrate.

# Supplementary Table S1

| **Supplementary Table S1. Demographic characteristics of patients receiving liver hemangioma resection or liver transplantation from whom liver samples were used in scRNA-seq.** | | | | | | |
| --- | --- | --- | --- | --- | --- | --- |
| **Group** | **Gender** | **Age** | **Height (cm)** | **Weight (kg)** | **BMI** | **Cirrhosis** |
| Ctrl | Male | 43 | 173 | 75 | 25.06 | No |
| Ctrl | Male | 46 | 175 | 75 | 24.49 | No |
| Ctrl | Female | 47 | 162 | 60 | 22.86 | No |
| LT | Male | 51 | 168 | 69 | 41.07 | Yes |
| LT | Male | 31 | 180 | 80 | 24.69 | Yes |
| LT | Male | 57 | 170 | 67.5 | 23.35 | Yes |
| Ctrl, patients with liver hemangioma resection; LT, patients with liver transplantation; BMI, body mass index. | | | | | | |

# Supplementary Table S2

| **Supplementary Table S2. Demographic characteristics of patients receiving liver hemangioma resection or liver transplantation from whom liver samples were used in RT-qPCR, western blotting, immunohistochemistry, and tissue Fe^2+^ measurements.** | | | | | | |
| --- | --- | --- | --- | --- | --- | --- |
| **Group** | **Gender** | **Age** | **Height (cm)** | **Weight (kg)** | **BMI** | **Cirrhosis** |
| Ctrl | Female | 55 | 158 | 61 | 24.44 | No |
| Ctrl | Male | 57 | 174 | 63 | 20.80 | No |
| Ctrl | Female | 47 | 162 | 60 | 22.86 | No |
| Ctrl | Male | 55 | 170 | 60 | 20.76 | No |
| Ctrl | Male | 39 | 168 | 60 | 21.26 | No |
| Ctrl | Female | 40 | 155 | 55 | 22.89 | No |
| LT | Male | 59 | 176 | 77 | 24.86 | Yes |
| LT | Female | 41 | 156 | 60 | 24.65 | Yes |
| LT | Male | 39 | 162 | 70 | 26.67 | Yes |
| LT | Male | 47 | 164 | 50 | 18.59 | Yes |
| LT | Female | 58 | 153 | 46 | 19.65 | Yes |
| LT | Male | 65 | 173 | 58 | 19.38 | Yes |
| Ctrl, patients with liver hemangioma resection; LT, patients with liver transplantation; BMI, body mass index. | | | | | | |
